# Pain, Agitation, Delirium, and Iatrogenic Withdrawal Syndrome Management in Children Who Are Critically Ill: Protocol for a European Clinical Practice Guideline Using the Grading of Recommendations Assessment, Development, and Evaluation Approach

**DOI:** 10.2196/67930

**Published:** 2025-09-08

**Authors:** Ibo MacDonald, Alexia Cavin-Trombert, Cécile Jaques, Gwenaëlle De Clifford-Faugère, Pieter A De Cock, Saskia N de Wildt, Dmytro Dmytriiev, Juliane Engel, Paola Claudia Fazio, Sylvia George, Isabelle Goyer, Anna Harðardóttir, Julia Harris, Klára Horváth, Erwin Ista, Santiago Mencía, Tuuli Metsvaht, Maria Cristina Mondardini, Mehdi Oualha, Maria-Helena Perez, Krzysztof Pietrzkiewicz, Francesca Sperotto, Benjamin Wyness, Nilüfer Yalındağ, ESPNIC Nursing and Allied Healthcare Professionals Section, ESPNIC Pharmacology Section, Angela Amigoni, Anne-Sylvie Ramelet

**Affiliations:** 1 Institute of Higher Education and Research in Healthcare Faculty of Biology and Medicine University of Lausanne Lausanne Switzerland; 2 Medical Library Lausanne University Hospital and University of Lausanne Lausanne Switzerland; 3 Department of Pharmacy Ghent University Hospital Ghent Belgium; 4 Department of Basic and Applied Health Sciences Ghent University Ghent Belgium; 5 Department of Pediatric Intensive Care Ghent University Hospital Ghent Belgium; 6 Department of Pharmacy, Pharmacology and Toxicology Radboud University Medical Center Nijmegen The Netherlands; 7 Department of Neonatal and Pediatric Intensive Care - Pediatric Intensive Care Erasmus MC – Sophia Children’s Hospital Erasmus University Medical Center Rotterdam The Netherlands; 8 Department of Anesthesiology and Intensive Care Vinnitsya National Medical University Vinnitsya Ukraine; 9 Intensive Care Medicine and Pulmonology Department of Cardiology University Children's Hospital Tuebingen Tuebingen Germany; 10 Pediatric Intensive Care Unit University-Hospital of Padua Padua Italy; 11 Paediatric Critical Care Unit & Department of Pharmacy Oxford University NHS Foundation Trust Oxford United Kingdom; 12 Department of Pharmacy, Pediatrics, Anaesthesia and Critical Care University Hospital of Caen Caen France; 13 Intensive Care Unit National University Hospital of Iceland Reykjavík Iceland; 14 Division of Children’s Nursing London South Bank University London United Kingdom; 15 Pediatric Center Semmelweis University Budapest Hungary; 16 Section Nursing Science Department of Internal Medicine, Erasmus MC Erasmus University Medical Center Rotterdam The Netherlands; 17 Pediatric Intensive Care Unit Gregorio Marañón Hospital Complutense University of Madrid Madrid Spain; 18 Institute of Clinical Medicine Department of Paediatrics University of Tartu Tartu Estonia; 19 Paediatric and Neonatal Intensive Care Unit Tartu University Hospital Tartu Estonia; 20 Pediatric Anesthesia and Intensive Care Unit Department of Woman's and Child's Health IRCCS AOUBO Bologna Italy; 21 Pediatric Intensive Care Unit Necker University Hospital Paris France; 22 Paris City University Paris France; 23 Paediatric Intensive and Intermediate Care Units, Service of Pediatrics Women-Mother-Child Department Lausanne University Hospital Lausanne Switzerland; 24 Faculty of Biology and Medicine University of Lausanne Lausanne Switzerland; 25 Department of Paediatric Anaesthesiology and Intensive Therapy Poznan University of Medical Sciences Poznan Poland; 26 Department of Cardiology Boston Children's Hospital Boston, MA United States; 27 Department of Pediatrics Harvard Medical School Boston, MA United States; 28 Pharmacy Department Cambridge University Hospitals NHS Foundation Trust Cambridge United Kingdom; 29 School of Medicine Marmara University Istanbul Turkey; 30 Division of Pediatric Critical Care Department of Pediatrics Marmara University Pendik Training and Research Hospital Istanbul Turkey; 31 See Acknowledgment; 32 Pediatric Intensive Care unit University Hospital of Padova Padova Italy

**Keywords:** assessment, comfort, critical care, pediatric intensive care, treatment

## Abstract

**Background:**

In pediatric intensive care units, pain, sedation, delirium, and iatrogenic withdrawal syndrome (IWS) must be managed as interrelated conditions. Although clinical practice guidelines (CPGs) exist, new evidence needs to be incorporated, gaps in recommendations addressed, and recommendations adapted to the European context.

**Objective:**

This protocol describes the development of the first patient- and family-informed European guideline for managing pain, sedation, delirium, and IWS by the European Society of Paediatric and Neonatal Intensive Care.

**Methods:**

This guideline will follow the Grading of Recommendations Assessment, Development, and Evaluation ADOLOPMENT approach across seven phases: (1) setup—establish 3 groups, namely a steering committee, development panel (experts and patient and family partners), and patient and family partner advisory panel, to define guideline scope through voting and consensus; (2) preparation—vote on 30 summary recommendations compiled from existing CPGs of medium quality or above; prioritize new research questions; update the search for CPGs to match new research questions with recommendations using population, intervention, comparator, and outcome elements; prioritize outcomes for effectiveness questions using a 9-point Likert scale; with validation from patient and family partners; (3) evidence identification, analysis, and data extraction—develop individualized search strategies for each research question (2 independent appraisers will select and appraise studies and conduct data extraction); (4) evidence synthesis—expert pairs will summarize findings in evidence profiles and evidence-to-decision (EtD) frameworks (in the absence of evidence, the expert panel will be surveyed to assess current practices); (5) guideline development—expert pairs will draft recommendations, then topic-specific subgroups will reach consensus before full development panel voting (>80% approval needed; subgroups will determine the need for additional supporting content); (6) review—conduct internal, society-level, and external international expert reviews using surveys with Likert scales and open-ended comments; and (7) issue and update—publish the guideline and monitor literature to assess the need for updates before 5 years.

**Results:**

In phase 1, a total of 21 clinical experts and 17 patient and family partners were recruited, and the guideline scope was finalized with 80% to 100% agreement. In phase 2, a total of 23 summary recommendations and 17 new research questions (total=40) were selected. The updated CPG search identified 2 low-quality CPGs, which were excluded from recommendation matching. Of the 17 new research questions, 4 matched existing recommendations. Of the 3 effectiveness questions, one had 7 prioritized outcomes, whereas two had 9 outcomes for inclusion in EtD frameworks. The final CPG is expected by spring 2026, with search strategies, EtD frameworks, and recommendations included.

**Conclusions:**

This protocol ensures a transparent Grading of Recommendations Assessment, Development, and Evaluation–based development process, leading to a trustworthy and credible guideline tailored to the European context for managing pain, sedation, delirium, and IWS in children who are critically ill.

**International Registered Report Identifier (IRRID):**

DERR1-10.2196/67930

## Introduction

### Background

Analgesia and sedation in children who are critically ill remain challenging for health care professionals in pediatric intensive care units (PICUs) due to the heterogeneous responses of patients to the same medications and the potential for drug side effects [1]. Suboptimal sedation can cause discomfort, agitation, and increased medication use [2]. Undersedation can increase anxiety, stress, and the risk of accidental medical equipment removal, including unplanned extubation [2,3], whereas oversedation may cause complications such as respiratory depression, hemodynamic instability, altered bowel function, increased morbidity risk, and prolonged PICU stay [2,4]. Prolonged use of analgesics and sedatives is associated with iatrogenic withdrawal syndrome (IWS) and delirium [5,6], both leading to long-term cognitive, emotional, and social impairments in children and causing significant distress for families [7,8]. Therefore, achieving optimal analgosedation levels in children who are critically ill is fundamental for their comfort and safety and requires close monitoring.

Accurate assessment is a prerequisite for appropriate management. The 2016 European Society of Paediatric and Neonatal Intensive Care (ESPNIC) recommendations emphasized the importance of using validated measurement instruments to monitor pain, depth of sedation, delirium, and IWS [9]. However, recent surveys have highlighted wide variations in assessment practices and choice of drugs for managing pain, sedation, delirium, and IWS (hereafter referred to as the 4 conditions) [10-12]. In addition, compliance with these recommendations has been inconsistent, with up to 69% of clinicians not selecting the correct measurement instruments, 30% misapplying them, and 3% to 58% not using them at all [13], leading to potential under- or overuse of medications and adverse events.

To improve assessment and management practices of pain, sedation, delirium, and IWS, clinical practice guidelines (CPGs) have been developed to synthesize evidence into evidence-based recommendations [14]. Since 2022, CPGs addressing these 4 conditions have been published [15,16]; however, newer pharmacological approaches and new evidence have emerged since then, necessitating a reassessment to determine the need for updated or newer recommendations. In addition, a recent narrative review identified 15 gaps in current CPG recommendations [17], further emphasizing the need for an updated CPG.

To ensure the highest quality and transparency of CPG development, it is essential that development panels follow a standardized methodology. The Grading of Recommendations Assessment, Development, and Evaluation (GRADE) approach is an internationally recognized standard for creating high-quality and transparent CPGs. In addition, guideline panels wishing to adopt or adapt recommendations from existing CPGs using the GRADE-ADOLOPMENT approach require the inclusion of clearly defined clinical questions and access to evidence-to-decision (EtD) tables [18,19]. These elements allow development panels to assess the credibility and relevance of existing recommendations before integration into a new CPG [18,19].

### Objectives

Given the emergence of new evidence, the necessity for European contextualization, and the importance of adhering to rigorous and transparent methodology, there is a need to develop an updated CPG. With the endorsement of ESPNIC and strict adherence to GRADE-ADOLOPMENT methodologies and reporting [18,19], we aim to develop a comprehensive and trustworthy European CPG for managing pain, sedation, delirium, and IWS in children who are critically ill.

## Methods

### Overview

The protocol for developing our CPG was registered in the Practice Guideline Registration for Transparency under registration number PREPARE-2024CN859 [20,21].

**Figure 1:**
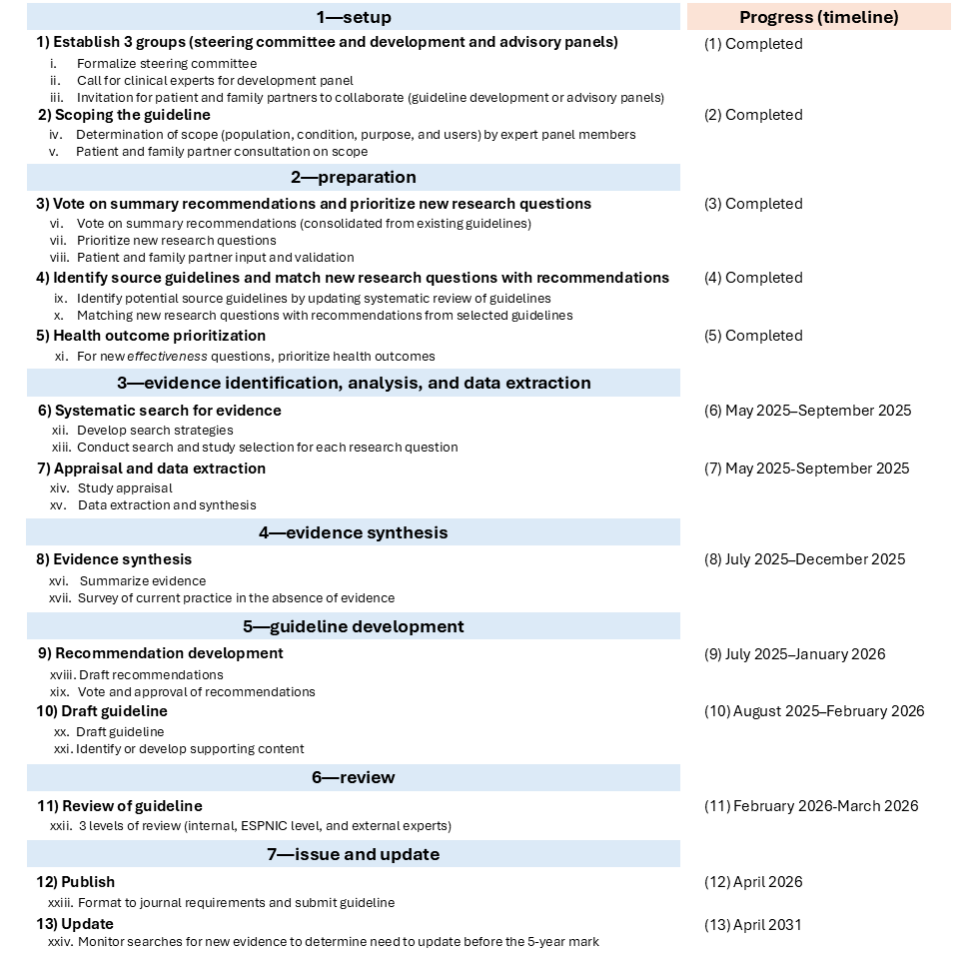
Phases, steps, and actions of the guideline development process, progress, and proposed timeline. ESPNIC: European Society of Paediatric and Neonatal Intensive Care.

The GRADE-ADOLOPMENT approach will be followed for guideline development, using a consensus-based process for each step and action, with prioritized research questions and outcomes [18,19]. The development process involving 7 phases, 13 steps, and 24 actions, along with the proposed timeline for completion, is summarized in Figure 1 and described in the following sections. As no reporting checklist exists for guidelines, one was developed based on the work by Xun et al [22] (Multimedia Appendix 1).

While this work is supported and endorsed by ESPNIC, none of the expert panel members receive payment for their contributions. The funding provided by ESPNIC will cover an annual subscription to the GRADEpro software (McMaster University and Evidence Prime Inc) and remuneration for patient and family partners for their time and may also support the librarian’s work on literature searches, with a total budget of €7160 (US $8174). ESPNIC will have no influence on the content of the CPG or forming recommendations, and no executive committee members will participate in voting activities.

### Phase 1: Setup

The setup phase comprises 2 steps and 5 actions.

#### Step 1: Establishing 3 Groups

In total, 3 groups will be formed: the steering committee, the development panel, and the patient and family partner advisory panel.

#### Action 1: Formalizing the Steering Committee

The steering committee includes a pediatric intensivist (AA), a PICU nurse and researcher (ASR), and a nurse methodologist (IMD). The steering committee will oversee, organize, and coordinate the development process. Committee members ASR and AA are institution supported and receive no funding for their work, whereas the chair (IMD) is supported by a postdoctoral fellowship for her role and will also take on all administrative support tasks. Invited guests to the steering committee will be the lead health sciences information specialists (AC-T and CJ), who will assist with planning and conducting the literature reviews.

#### Action 2: Call for Clinical Experts for the Development Panel

The development panel will include clinical experts and patient and family partners (the recruitment of all patient and family partners is explained in action 3).

In April 2023, a call for expressions of interest to join the development panel was sent to the Analgosedation CONSORTIUM from the Pharmacology Section and the Nurse Science Section of ESPNIC, and an open invitation was extended to participants at the June 2023 ESPNIC conference. Interested members completed a declaration of interest and conflict of interest (COI) form (Multimedia Appendix 2). The expert panel will include nurses, intensivists, and pharmacists from as many European countries as possible who will volunteer their time.

#### Action 3: Invitation for Patient and Family Partners to Collaborate

Clinical expert panel members will identify families or patients from their existing networks who have experience with one or more conditions covered in the CPG. Identified individuals will be provided with a patient and family partner information sheet outlining the purpose and description of involvement across the 2 groups, as well as an expression of interest form that includes a COI declaration (Multimedia Appendix 3) [23-27]. Interested patients and family members will be able to choose to join either the development panel or the patient and family partner advisory panel depending on their level of comfort with the English language and ability to engage regularly. They will indicate their group preference on the expression of interest form.

Children will be included in the patient and family partner advisory panel to represent the pediatric perspective in a consultative capacity. With parental support, children can provide their insights based on their experiences. Parental consent will be provided for all children, and the steering committee lead will only contact them through their parents. Children aged 12 years will be invited to participate in patient and family partner advisory panel activities such as surveys or interviews as they have the capacity for health-related decision-making at this age [28]. Before engaging, assent will be obtained from each child to ensure that they understand the activity and their role with parental presence. For any activity, their role and participation are entirely voluntary. All data and experiences will be anonymized to maintain confidentiality. Infants will be represented through their parents or carers, who will provide input based on their experiences with the child in the PICU.

For all patient and family partner activities, if translation of forms or surveys is required, an established translation method will be used [29]. This involves an initial translation using online software followed by verification by a bilingual clinical expert who is a native speaker to ensure accuracy. Given the multiple languages involved, all meetings will be held on the web, allowing participants to enable captioned or real-time translation as needed. All meetings will be recorded and stored on a web-based project management platform where all members will have access to all materials.

The roles, responsibilities, and level of engagement and involvement across the 3 groups are outlined in Table 1.

**Table 1:** Roles and responsibilities of the 3 groups.

			Steering committee		Development panel		Patient and family partner advisory panel
Role			Oversees and coordinates the guideline’s development and ensures that all preparatory tasks are completed		Extensive discussions to draft, revise, and formalize the guideline		Provides patient and family perspectives, confirms decisions, and reviews the guideline
**Responsibilities**							
	Scope	Draft		Establish		Advise and validate	
	Vote on summary recommendations	No		Yes		Advise and validate	
	Prioritizing new research questions	No		Yes		Advise and validate	
	Prioritizing outcomes for effectiveness questions	No		Yes		Yes	
	Updating systematic review of guidelines	Yes		No		No	
	Matching research questions to recommendations	Yes		No		No	
	Updating searches	Yes		Yes		No	
	Screening titles and abstracts	Yes		Yes		No	
	Full-text review	Yes		Yes		No	
	Appraisal of studies	Yes		Yes		No	
	Data extraction	Yes		Yes		No	
	Creating evidence tables	Draft		Pairs—draft, review, and revise		Review and provide input from a patient and family perspective	
	Formulating recommendations	Draft		Pairs—draft; subgroups—revise		Provide input and confirm	
	Vote on drafted recommendations	No		Yes		Yes	
	Writing the guideline	Draft		Pairs—draft; subgroups revise		No	
	Creating accompanying materials	Draft		Draft		Possibly draft	
	Reviewing the guideline	Yes		Yes		Yes if able	

All members of the development panel will complete a COI form covering financial, academic, clinical, and community aspects [30]. At the beginning of each development panel meeting, the steering committee chair will ask whether any members have changes to their COIs to declare. If yes, a new COI form will be completed. The steering committee will monitor COIs; members with a declared COI will not participate in the development or voting on recommendations related to their COI.

Withdrawing from participation in any group is acceptable at any stage of the development process and for any reason without the need for disclosure. Patient and family partners should inform the chair if they choose to withdraw but may also simply stop responding to requests (eg, surveys). Expert panel members must notify the chair if they decide to leave the development panel. While there is no formal process for expelling members due to inappropriate behavior, this will be left to the discretion of the steering committee.

Training will be provided either during panel meetings or through available online resources [31-33].

Clinical expert panel members will have their information presented in a table, including all relevant information (ie, organization, geographic location, role on the development panel, and COI) as specified in item 3 of the Appraisal of Guidelines for Research and Evaluation (AGREE) II instrument [34]. The basic characteristics of patient and family partners and PICU experiences of children will be presented in 2 separate tables.

#### Step 2: Scoping the Guideline

##### Action 4: Determining the Scope

The population, conditions, purpose, and users of the CPG will be determined during the first development panel meeting with clinical experts through consensus-based discussions.

##### Action 5: Patient and Family Partner Consultation on Scope

The results of action 4 will be sent to all patient and family partners for review, input, and validation using SurveyMonkey (SurveyMonkey Inc) [35]. Patient and family partners will indicate whether they “reject,” “accept,” or “accept with modifications” in each area, indicating any desired changes. A percentage for acceptance or rejection will be calculated for each area, with consensus defined as ≥80% for confirmation. The steering committee will review and revise as needed and will present any modifications at the next scheduled development panel meeting, where a final consensus of 80% agreement of participating clinical experts will be needed.

The results of scoping will be presented in a table outlining the progression from clinical expert drafting to patient and family partner consultation to final consensus.

#### Phase 2: Preparation

**Figure 2:**
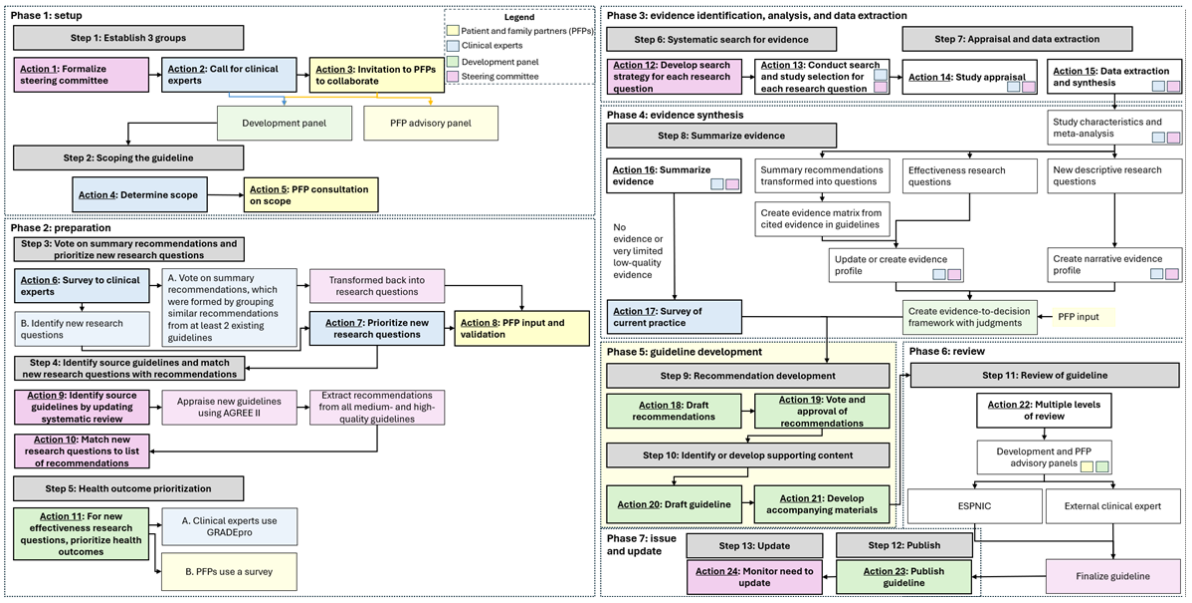
Guideline development flowchart. AGREE: Appraisal of Guidelines for Research and Evaluation; ESPNIC: European Society of Paediatric and Neonatal Intensive Care; PFP: patient and family partner.

The preparation phase includes 3 steps and 6 actions. To help illustrate the development process, Figure 2 provides the development flowchart.

#### Step 3: Voting on Summary Recommendations and Prioritizing New Research Questions

##### Action 6: Vote on Existing Summary Recommendations

The 30 summary recommendations as synthesized from common recommendations found across multiple existing CPGs of medium and higher quality, as identified in a systematic review [36]. These serve as a preliminary set of recommendations reflecting current practices and evidence to establish a baseline set of “included recommendations.”

The voting process will use the GRADE-ADOLOPMENT step [18], whereby the summary recommendations will be added to SurveyMonkey [35] and each clinical expert will vote to “accept (adopt),” “reject,” or “accept with modifications (adapt)” each summary recommendation, specifying modifications if needed. Responses will be tallied, with “accept” and “accept with modifications” grouped together. A consensus of ≥80% will be needed for inclusion. Summary recommendations that fail to meet this threshold will be discussed during a consensus meeting with the development panel, where a final decision will be made. The survey will also include an open-ended question asking about clinical areas in which new research questions are needed (action 7). Initially, clinical experts will vote, whereas all patient and family partners will provide input later (action 8).

##### Action 7: Prioritizing New Research Questions

A list of research questions will be created based on the gaps identified by Mondardini et al [17] and areas without recommendations from the earlier survey (action 6). These questions will be entered into the GRADEpro Guideline Development Tool (GDT), a web-based tool that helps development panels brainstorm, prioritize questions and outcomes, and create evidence profiles and summary of findings tables [37].

The prioritization process in the GRADEpro GDT involves the following steps:

1. Consolidation and reformulation—the steering committee will consolidate the questions by removing duplicates and, where possible, reformatting them into population, intervention, comparator, and outcome effectiveness questions [38]. Nonactionable or descriptive questions will also be included.
2. Brainstorming—clinical experts will independently review and comment on the questions and suggest new questions if needed.
3. Second consolidation—the steering committee will refine and reformat questions from the brainstorming round to prepare for voting.
4. Voting—clinical experts will independently rate the importance of each question on a 9-point Likert scale (1-3=nonimportant, 4-6=important, and 7-9=critically important). The GRADEpro GDT will calculate mean scores and rank the questions from highest to lowest [39].
5. Decision-making—the steering committee will review the voting results and decide the outcome of each question—reject (mean rating of 1-3), answer (mean rating of 7-9), or list for potential inclusion in future CPGs (mean rating of 4-6).
6. Approval—clinical experts will review the refined list of questions and comment if not approved. GRADEpro GDT will generate a matrix showing the percentage of approval for each question [39]. These results will be shown at the next development panel meeting, where clinical experts will discuss and approve all final questions for inclusion in the guideline. Questions with >80% agreement will be retained.
7. Final decisions—a member of the steering committee will enter the approval questions into GRADEpro GDT. For any effectiveness questions, evidence profiles will be developed directly in the GRADEpro GDT.

##### Action 8: Patient and Family Partner Input and Validation

The final list of summary recommendations (action 6) and retained new research questions (action 7) will be sent to patient and family partners using SurveyMonkey [35]. They will confirm their agreement by selecting “yes,” “no,” or “yes with modification.” Patient and family partners can also propose new questions if they believe any are missing. A consensus of ≥80% is needed to retain questions and recommendations. The steering committee will review and revise based on suggested modifications, and the results will be discussed during the next development panel meeting with clinical experts.

The results of step 3 will be presented as a pair of tables that outline the clinical expert, patient and family partner, and consensus meeting results for both summary recommendations and new research questions.

##### Step 4: Identifying Source Guidelines and Matching New Research Questions With Recommendations

The cornerstone of the GRADE-ADOLOPMENT approach is adopting and adapting recommendations from existing CPGs by matching them to questions [18]. This process involves evaluating the relevance, applicability, and strength of the original recommendations [18].

##### Action 9: Identifying Potential Source CPGs by Updating the Systematic Review of CPGs

To ensure an up-to-date list of recommendations for matching the prioritized new questions from action 7, the systematic review of CPGs for managing the 4 conditions will be updated following the same methods used previously [36] with two changes. First, 2 appraisers instead of 3 will be used. Second, they will review their results for consensus to ensure that no important criteria elements are missed during the appraisal, thereby minimizing the risk of skewed scoring when reducing to 2 appraisers, which is the minimum number recommended when using the AGREE II tool [34]. If domain 3 (rigor of development) is >60% (calculated as a percentage of the maximum possible score for each domain, based on consensus between reviewers), the CPG will be retained, and its recommendations will be used for matching. The search results will be presented using a PRISMA (Preferred Reporting Items for Systematic Reviews and Meta-Analyses) flow diagram [40] and a table of the AGREE II consensus scores.

##### Action 10: Matching New Research Questions With Recommendations From Selected Guidelines

The steering committee members will assess whether any new research question matches recommendations from the 6 medium- and higher-quality CPGs [9,15,16,41-43] from our systematic review [18] or from any CPGs identified during the update of this systematic review (action 9). All recommendations have been extracted from the preselected CPGs; if additional CPGs are identified, 1 steering committee member will extract these and add them to the list. The same steering committee member will then assess each new research question against this list of recommendations to determine whether there is a match. A match is based on the population, intervention, and comparator and at least one shared outcome matching. After initial matching, the other 2 steering committee members will verify all matches, and consensus must be reached through discussion among all 3 members. The results of this action will be presented in a table outlining the progression from matching to consensus.

#### Step 5: Health Outcome Prioritization (Action 11: Health Outcome Prioritization)

This step focuses on prioritizing health outcomes for unmatched new research questions that are related to effectiveness. This excludes any new research questions that are nonactionable or descriptive.

In total, 2 simultaneously administered methods will be used to prioritize health outcomes, one for clinical experts using the GRADEpro GDT (the process will mirror that of action 7) and one for patient and family partners using SurveyMonkey [35]. For clinical experts, the process will be as follows: (1) brainstorming—the steering committee will generate a list of important health outcomes for the PICU from the literature [36,44-46], health outcomes will be assigned to each effectiveness question, and clinical experts will independently review these and suggest others; (2) condense—the steering committee will review and consolidate health outcomes as needed; and (3) voting—clinical experts will independently rate the importance of each health outcome for each question using a 9-point Likert scale (1-3=nonimportant, 4-6=important, and 7-9=critically important).

For patient and family partners, a survey will be created using SurveyMonkey [35] that includes each effectiveness question along with the outcomes identified from the PICU literature [36,44-46]. These will be sent to patient and family partners, and they will independently rate the importance of each health outcome for each question using a 9-point Likert scale (1-3=nonimportant, 4-6=important, and 7-9=critically important), along with adding and rating any missing health outcomes.

The fourth step in this process is decision-making (from this step onward, the results of both groups are used). The steering committee will review the voting results from both groups together to decide which health outcomes to reject (mean rating of 1-3), include in EtD tables (mean rating of 7-9), or list in the body or text (mean rating of 4-6) for each effectiveness question. A maximum of 7 health outcomes is recommended for inclusion in EtD tables per question [47,48]. The steering committee will include the top 5 health outcomes from both prioritizing activities (assuming some overlap). If more than 7 health outcomes are identified as critically important (mean rating of 7-9), all of them will be included. The fifth step is approval. The results will be presented to the clinical experts at the next development panel meeting for final approval (≥80% agreement is needed for consensus).

The results of this step will be presented in a table outlining the progression from prioritization to consensus across both groups.

### Phase 3: Evidence Identification, Analysis, and Data Extraction

#### Step 6: Systematic Search for Evidence

**Figure 3:**
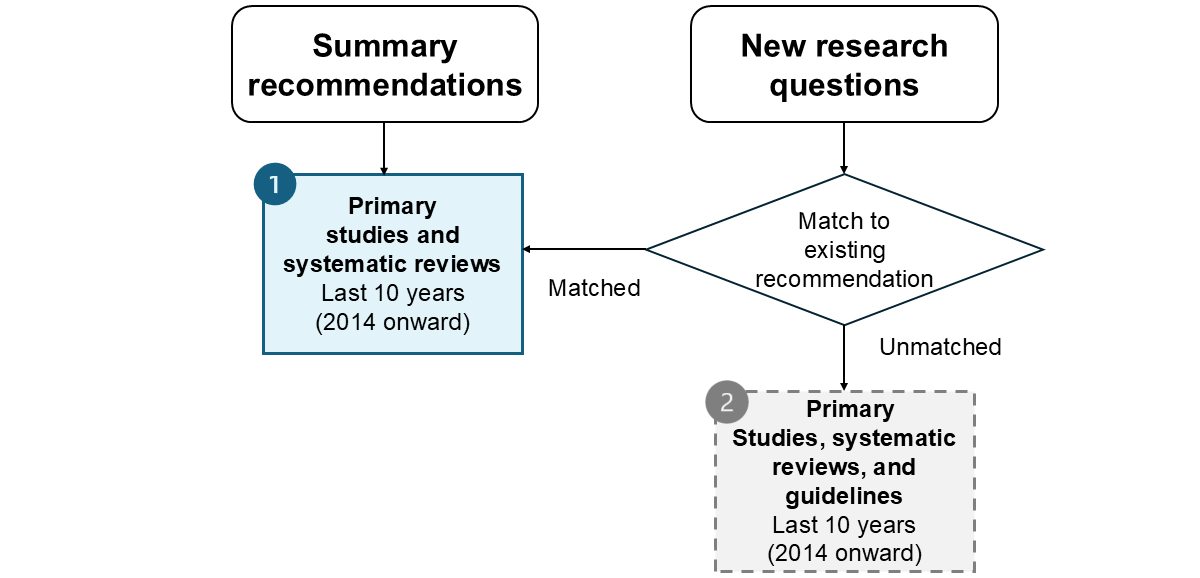
The 2 search approaches.

This phase involves systematic searching for evidence using 2 distinct search approaches, one for summary recommendations (search approach 1) and one for new research questions (search approach 2), as illustrated in Figure 3. Summary recommendations will be transformed back into research questions to aid in developing a search strategy for each.

#### Action 12: Development of Search Strategies

For summary recommendations a search for primary studies and systematic reviews will be conducted (search approach 1). CPGs are omitted as summary recommendations originate from CPGs.

For new research questions a combined search for primary studies, systematic reviews, and CPGs will be conducted [49].

Both search approaches will limit the results to the last 10 years to ensure up-to-date evidence.

Searches will be conducted in 4 electronic databases for both search approaches: MEDLINE ALL (Ovid), Embase, CINAHL with Full Text (EBSCO), and Epistemonikos.

For approach 2, the same guideline repositories and society websites used in our systematic review [36] will be searched to identify relevant CPGs.

To update the evidence for each summary recommendation and answer each new research question, a customized search strategy will be developed by the steering committee (IMD) in consultation with a health sciences librarian (ACT or CJ) using index and free terms describing the conditions of interest and the population of interest (children aged 0 to 18 years), and the comparison, outcome, and intervention will be adapted for each. Another health services librarian will peer review each search strategy using the Peer Review of Electronic Search Strategies checklist [50]. All search strategies will be included in the supplementary material of the published CPG.

No language restrictions will be applied; non–English-language studies will be translated using a standardized method [29]—DeepL Translator (DeepL SE) [51] to translate and then verified by a native speaker on the development panel or found via panel members’ networks. If one cannot be found, the study will not be included.

#### Action 13: Conducting the Search and Study Selection for Each Research Question

For each search (summary recommendations and new research questions), retrieved records will be imported into EndNote (version 20; Clarivate Analytics), and duplicates will be removed using the Deduklick automated algorithm (Risklick) [52]. Screening and full-text review will be independently conducted by 2 reviewers using Rayyan (Qatar Computing Research Institute) [53]. Disagreements will be resolved through consensus or by a third reviewer. For each research question, the selection process will be documented using a PRISMA flowchart [40] and a table of excluded studies, which will be included in the supplementary materials of the published CPG.

#### Step 7: Appraisal and Data Extraction

##### Action 14: Study Appraisal

Appraisal will be conducted based on study type—systematic reviews, primary studies, or CPGs—by 2 independent appraisers.

For summary recommendations, all the referenced studies from the source guidelines will be extracted. One member of the steering committee will evaluate each reference for relevance to and support for the recommendation using a 4-point Likert scale (1=*extremely*; 4=*not at all*) to determine whether the study should continue to be included for the development of the evidence profile (action 16; Multimedia Appendix 4, Table 1).

For all identified studies (including those from summary recommendations and new studies identified from searches in action 13), the following appraisal tools will be used.

For systematic reviews, quality will be assessed using A Measurement Tool to Assess Systematic Reviews 2, a 16-item tool that evaluates overall quality, ranging from critically low to high based on the presence of critical flaws [54].

The JBI checklists will be used to assess the quality of primary studies—randomized controlled trials [55], quasi-experimental studies [56], qualitative research [57], prevalence studies [58], cohort studies [59], economic evaluations [60], diagnostic test accuracy studies [61], case reports [62], case-control studies [62], and analytical cross-sectional studies [62]. Each appraiser determines the level of bias for each item, including a decision to “include,” “exclude,” or “requires more information.”

All systematic reviews and primary studies will be retained regardless of quality, and results will be analyzed accordingly, with higher-quality studies given more weight and systematic reviews prioritized over primary studies.

The AGREE II, a 23-item validated appraisal instrument, will be used to evaluate the quality of CPGs using a 7-point Likert scale across 6 domains [34]. Based on consensus between reviewers, each domain will be scored as a percentage of the maximum possible score, if domain 3 (rigor of development) scores >60%, the CPG will be retained.

The results of all appraised studies will be presented in tables describing the characteristics of the included studies for each research question.

The literature will continue to be monitored via monthly saved searches and added up until the development panel completes its final voting on recommendations (action 19).

##### Action 15: Data Extraction and Synthesis

One person will extract data from each study, and this will be verified by another person using a predefined Microsoft Word document that includes descriptive information on the study characteristics, health outcome results, and any patient and family perspectives (qualitative or quantitative; Multimedia Appendix 4, Table 2). This will be pilot-tested by 2 members, and any changes will be reported in the published CPG.

For effectiveness questions, health outcomes will be extracted as means for potential meta-analysis if at least 3 studies exist with the same health outcome data. If medians and IQRs are reported, the method by Wan et al [63] and the Microsoft Excel tool will be used to transform them.

Meta-analyses will be conducted using the Stata software (version 17; StataCorp) [64]. Random-effects models applying the Sidik-Jonkman method will be used with the standardized mean difference for continuous variables or odds ratios for dichotomous variables. The *I*^2^ test will be used to assess statistical heterogeneity, with values of 40% considered low, values of 30% to 60% considered moderate, values of 50% to 90% considered substantial, and values of 75% to 100% deemed considerable [65]. If heterogeneity is ≥40%, a sensitivity analysis will be conducted by removing one study at a time to assess its influence on the overall effect size [65]. In addition, sensitivity analysis will be conducted on population, intervention, comparator, and outcome elements; study design; or risk of bias if sufficient studies (>2) are available to explore other sources of heterogeneity. The results will be presented as forest plots and funnel plots, with the Egger test used to assess publication bias, especially type I error [66]. If a meta-analysis is not possible, the results will be described narratively.

### Phase 4: Evidence Synthesis

#### Step 8: Evidence Synthesis

##### Action 16: Summary of Evidence

The GRADE approach focuses on using the best available high-quality evidence, typically from systematic reviews [65]. To do this, 2 types of GRADE evidence tables [67] will be created.

The first type is evidence profiles, which provide information about the body of evidence and the judgments made about the quality of the evidence, included as statistical results for each outcome, per question [67]. These are completed at the end of phase 3 by 2 reviewers using the GRADEpro GDT for new effectiveness questions. The categories addressed by these tables include outcomes, number of studies, study design, quality of evidence factors, risk, effect, and overall quality of evidence (high, moderate, low, or very low). Evidence quality is determined by reviewing 5 factors that can lead to downgrading (risk of bias [37], inconsistency [68], indirectness [69], imprecision [70], and publication bias [71]) and 3 factors that can lead to upgrading (a large magnitude of effect, a dose-response gradient, and when all plausible confounders would increase the confidence in the effect [65,72,73]; Multimedia Appendix 5, Table 1).

These are used to create the EtD framework, which is the second type of evidence table. These simplified tables summarize the evidence profile without judgment details and will include information on other factors such as health benefits and harms, equity, feasibility, acceptability, and cost [18,67] (Multimedia Appendix 5, Table 2 [74]).

For nonactionable or descriptive questions, narrative evidence profiles will be created using the Etd framework table but merging the column containing the summary of findings and describing the results of the studies.

The steering committee will assign 2 clinical experts from the development panel to each summary recommendation and new research question (hereafter collectively referred to as “research question”) based on their domain of expertise to complete both tables. Once completed, the EtD framework will be sent to patient and family partners for feedback on any missing elements related to their experiences. Following reception of this feedback, the pair of clinical experts will complete the summary judgment table based on the GRADEpro GDT [39] (Multimedia Appendix 5, Table 3).

##### Action 17: Survey of Current Practice in Absence of Evidence

If search results for a specific research question reveal little to no supporting evidence, the steering committee will develop a survey with open-ended questions to gather information on current practices from clinical experts on the development panel, emphasizing cases and outcomes [75,76]. The results will be included in an EtD framework.

### Phase 5: Guideline Development

#### Step 9: Recommendation Development

The recommendation development process will include several steps within each action.

##### Action 18: Drafting of Recommendations

The first step is drafting the recommendations. Paired clinical experts will draft recommendations based on the evidence profile, EtD framework, and summary judgment tables using the recommendation table based on the GRADEpro GDT [39] (Multimedia Appendix 6). Recommendations will be categorized as *strong* (desirable effects outweigh undesirable effects, and the recommendations are applicable to almost all patients), *conditional* (desirable effects probably outweigh undesirable effects, and the recommendations are applicable to most patients but sensitive to preferences), or *good practice statements* (highly relevant, with benefits far outweighing harms even with indirect supporting evidence [all surveys of current practice with clinical experts will automatically be considered as a good practice statement; action 17]) [72]. The recommendation table includes the recommendation, its direction (for or against), its strength (recommend or suggest), balance of consequences, justification, considerations for subgroups (different populations or conditions), implementation considerations, monitoring and evaluation criteria, and identified research gaps.

Recommendations for any accompanying documents or materials will be sent to the steering committee (ie, resources for implementation, patient information sheet, and monitoring criteria).

The second step is subgroup review. The steering committee will form subgroups focused on pain, sedation, delirium, IWS, and “other” topics, with each subgroup including pairs assigned to specific research questions within the topic area. Subgroups will meet to review, revise, and update the recommendation table for all their assigned research questions. A steering committee member will be present to address methodology questions. The goal is to reach consensus of at least 80% on the wording, direction, and strength of each recommendation. If consensus is not reached, the recommendation will be discussed at the next development panel meeting.

In the published CPG, all accompanying recommendation tables, along with results from the consensus steps, will be provided by research question in the supplementary materials.

##### Action 19: Vote and Approval of Recommendations

The first step is recommendation approval voting. For each recommendation, the related tables (evidence profiles, EtD framework, summary of judgment, and recommendation) will be sent in one document to all development panel members for review at least 2 weeks before the next planned panel meeting. During the meeting, members will vote to “accept,” “reject,” or “accept with modifications.” At least 80% of panel members must be present to commence voting, with 80% consensus required for approval on each recommendation. Recommendations not reaching 80% approval will require further subgroup revision and voting until 80% approval is achieved.

The second step is recommendation review by the patient and family partner advisory panel. The final list of approved recommendations will be sent to the patient and family partner advisory panel using SurveyMonkey [35] for review and comment. They will be asked to “accept,” “reject,” or “accept with modifications” each recommendation.

The third step is recommendation finalization. The steering committee will make changes based on any suggested modifications. An update and discussion will take place at the next scheduled meeting, followed by consensus to approve any recommendation modifications.

All consensus results of voting will be documented and provided in the final publication of the CPG.

#### Step 10: Drafting the Guideline and Accompanying Content

##### Action 20: Drafting the Guideline

Once recommendations are finalized, each recommendation pair will draft their section for the CPG. This will then be reviewed by the entire subgroup before sending to the steering committee. The steering committee will compile, standardize, and expand (if needed) each recommendation.

##### Action 21: Identifying or Developing Supporting Content

The steering committee will convene to review all suggestions for supporting materials from panel pairs (the first step of action 18) and make decisions on what is needed and feasible. Necessary materials will be brought to a development panel meeting for discussion, and work will be distributed to subgroups or patient and family partners. Subgroups will first identify any readily available resources online and verify their copyright status. If freely available to use, they will be included in a compiled list of resources supporting that recommendation. Once finalized, the subgroup will then distribute this list, and each subgroup member will assess the suitability of each resource based on the target audience, level of language, number of languages available, and relevance to the recommendation using a 5-point Likert scale. The results will be tabulated, and the subgroup will meet to reach a consensus on which resources to recommend as supporting material. If no suitable resources are identified, the subgroup will determine whether new materials should be developed.

Supporting content will be included either under a subheading in the main text related to each recommendation or under the implementation and monitoring section of the published CPG, or as supplementary materials, depending on the type of content.

### Phase 6: Review (Step 11: Review of the CPG and Action 22: 3 Levels of Review)

The draft CPG will undergo three separate and sequential reviews: (1) internal review by the development panel and the patient and family partner advisory panel, (2) consultation open to all ESPNIC members, and (3) external review by invited international experts identified by the steering committee and development panel.

These reviews will be conducted through a web-based survey using SurveyMonkey [35], with a link sent via email from the steering committee chair using the ESPNIC internal distribution list and personal contacts. Reviewers will evaluate the CPG for overall agreement and clarity by section using a 9-point Likert scale. Each section will have a comment area for missing information, suggestions for modifications, setting-specific issues, and implementation implications.

The steering committee members will compile information on those who reviewed and their feedback from each review stage and revise the draft accordingly. Any major change that substantially alters the CPG will be discussed at a development panel meeting, with changes requiring 80% approval. The changes and review will be documented and appended to the final CPG.

### Phase 7: Issue

#### Step 10: Publishing (Action 23: Format to Journal Requirements and Submission of Guideline)

The CPG development process will culminate in its publication in a peer-reviewed journal. The steering committee will format the final CPG using the instructions for authors from the target journal. The RIGHT-Ad@pt checklist [77] will be used to ensure the transparent reporting of the adoption process, and the AGREE II instrument will be used to ensure the quality and reporting of the CPG [34] The target date for completing this CPG is by the end of spring 2026.

#### Step 11: Updating (Action 24: Monitoring Searches for New Evidence to Determine the Need to Update Before the 5-Year Mark)

As each recommendation will have an established search strategy, an annual review of new publications will be conducted by the guideline lead. If new evidence emerges that could change a recommendation from conditional to strong, the steering committee will be convened to assess whether an update is warranted. Otherwise, new evidence will be continuously monitored and accumulated, and at the 5-year mark, an update will commence.

### Ethical Considerations

The CPG development process is focused on transparency, integrity, and respect for all contributors. As neither clinical experts nor patient and family partners will undergo medical procedures or be required to follow specific rules of behavior as defined by the Medical Research Involving Human Subjects Act [78], formal institutional ethical review board approval was deemed unnecessary. While formal ethics submission is not required, all clinical experts and patient and family partners will complete COI forms to ensure impartiality during the development process. In addition, patient and family partners will provide informed consent for their participation in any patient and family partner advisory panel activities, such as surveys collecting patient and family partners’ experiences and perspectives, to ensure their participation is voluntary and that their role in shaping the CPG is acknowledged. All surveys are anonymized to protect privacy and confidentiality.

Regarding compensation, clinical experts volunteer their time and are not compensated. Patient and family partners on the development panel will be compensated at an hourly rate (€60 [US $70]) for their participation in panel meetings. For tasks outside of meetings, such as voting and reviewing, all patient and family partners will receive a flat-rate compensation of €90 (US $103). These compensation rates were determined by averaging rates used by represented countries and by referring to recommendations from the National Institute for Health and Care Research [79].

## Results

The setup and preparatory phases of the guideline development process have been completed, with the results described in the following sections.

### Phase 1: Setup

#### Step 1: Establishing Groups (Steering Committee and Development and Advisory Panels)

##### Action 1: Formalizing the Steering Committee

The 3-member steering committee was formalized before the writing of this protocol and was approved during the first online meeting of clinical expert members in February 2024.

##### Action 2: Call for Clinical Expert Members

Following calls for participation from members of the Analgosedation CONSORTIUM from the Pharmacology Section and the Nurse Science Section of ESPNIC and at the ESPNIC 2023 congress, 21 clinical experts from 14 countries submitted expressions of interest to join the development panel and completed their COI forms. A table listing all clinical experts, including their names, institution, location (city and country), professional designation, areas of expertise, and any identified COIs, is included in Multimedia Appendix 7 (patient and family partners not included).

##### Action 3: Invitation for Patient and Family Partners to Collaborate

Clinical experts began reaching out to potential patient and family partners in May 2024. By March 2025, 17 patient and family partners completed expression of interest forms, including COI disclosure. This group consisted of 76% (13/17) parents (with 2 families involving both parents choosing to participate, totaling 4 individuals) and 24% (4/17) children aged 6 to 16 years. These patient and family partners represent 6 countries. The characteristics of the patient and family partners are detailed in Table 2, whereas the characteristics of the children who have experienced the PICU are detailed in Table 3. No COI were reported by any patient and family partners.

**Table 2:** Characteristics of patient and family partners who submitted expressions of interest.

Characteristic			Patient and family partners		
			Parents (n=13), n (%)		Children (n=4), n (%)
**Sex**					
	Female	9 (69)		0 (0)	
**Country**					
	Switzerland	4 (31)		2 (50)	
	United Kingdom	4 (31)		0 (0)	
	Italy	2 (15)		1 (25)	
	Hungary	1 (8)		1 (25)	
	Spain	1 (8)		0 (0)	
	Turkey	1 (8)		0 (0)	

**Table 3:** Characteristics of the children who experienced the pediatric intensive care unit (PICU; N=12).

Characteristics and categories		Children
**Sex, n (%)**		
	Female	8 (67)
**Medical reason for admission, n (%)**		
	Respiratory	4 (33)
	Surgery	3 (25)
	Cardiac	2 (17)
	Sepsis	1 (8)
	Inflammatory syndrome	1 (8)
	Cancer	1 (8)
Supported on mechanical ventilation, n (%)		11 (92)
**Experience with conditions, n (%)**		
	Delirium	7 (58)
	Iatrogenic withdrawal syndrome	8 (67)
Age (y), mean (SD)		7.5 (6)
Time in PICU (d), mean (SD)		262 (515)
Time on mechanical ventilation (h), mean (SD)		11,716 (2740)

#### Step 2: Scoping the Guideline

##### Action 4: Determining the Scope

During the first meeting with clinical experts, an initial scope was drafted (second column of Table 4).

**Table 4:** Evolution of scope based on consensus rounds.

Scope elements	Initial draft	Patient and family partner suggestions for modification	Development panel discussion and decision	Final decision
Purpose	To develop a high-quality, evidence-based clinical practice guideline tailored to the European context. This guideline will incorporate the perspectives of patients, families, and health care professionals, aiming to improve the management of the interrelated conditions of pain, sedation, delirium, and IWS^a^ for children who are critically ill in PICUs^b^ by providing actionable, trustworthy, and credible recommendations.	Including children in intermediate care and children hospitalized for oncological diseases	As the focus was on children who are critically ill in the PICU, it was determined that these suggestions were already encompassed within the purpose. It was slightly rearranged for ease of comprehension.	To develop a high-quality, evidence-based guideline informed by the perspectives of patients, families, and health care professionals to improve the management of the interrelated conditions of pain, sedation, delirium, and IWS in children who are critically ill. This guideline aims to provide actionable, trustworthy, and contextually relevant recommendations for health care professionals caring for children who are critically ill in PICUs (primary setting), but the guideline could also be applicable to health care professionals managing children who are critically ill in settings such as the emergency department (awaiting transfer) and intermediate care unit to support continuity and consistency of care.
Population	Children who are critically ill aged 0 to 18 y in PICUs as the primary setting, but including those awaiting admission (eg, emergency department or during transport)	It could also include children being on a temporary home leave directly from (curated by) the PICU (eg, with a provided oxygen ventilator machine or oxygen cylinder) Including children in intermediate care and children hospitalized for oncological diseases	While other settings or conditions may be important, the focus is on children who are critically ill in the PICU. These additional populations and settings were not included. In addition, to ensure clarity regarding included population, excluded populations were added.	Children who are critically ill aged 0 to 18 y admitted for any medical reason to the PICU as the primary setting, but it can also include children who are critically ill in the emergency department (awaiting transfer) and the intermediate care unit. Patients receiving palliative or end-of-life care, neonates in neonatal intensive care units, and adults are excluded.
Users	This guideline is intended for health care professionals working in PICUs, including physicians, nurses, pharmacists, and other relevant allied health practitioners. It will also serve as a resource for patients, families, and caregivers.	Including health care professionals in intermediate care and pediatric oncology (children undergoing oncology treatment can also face these issues, for example, in the event of medication administration errors)	Not aligned with the focus on staff working in the PICU More precision needed to define the “other health care professionals”	This guideline is intended for health care professionals involved in the care of children who are critically ill in PICUs, including physicians, nurses, pharmacists, and other health care professionals. Depending on the patients’ needs and clinical status, other relevant health care professionals include physiotherapists, occupational therapists, respiratory therapists, physician assistants, psychologists, or child life specialists. The guideline will also serve as a resource for patients, families, and caregivers.
Conditions covered	Pain, sedation or agitation, delirium, and IWS	None	None	No change

^a^IWS: iatrogenic withdrawal syndrome.

^b^PICU: pediatric intensive care unit.

##### Action 5: Patient and Family Partner Consultation

A survey was conducted among all patient and family partners in February 2025 to review and comment on the 4 drafted scope elements. In total, 10 of the eligible 16 patient and family partners completed the survey (63%), with agreement on the 4 elements ranging from 80% to 100% acceptance (Multimedia Appendix 8 details the agreement with scope elements).

Some modifications were suggested by patient and family partners, among them the inclusion of patients with cancer and the intermediate care unit, which was repeated across multiple scope elements. These suggestions were discussed with the clinical experts at the next meeting. It was agreed that the primary focus is on critically ill children in the PICU. Additional populations such as patients with cancer would not be included as they fall outside the primary focus. However, the intermediary care unit was added, with the emphasis being that the CPG applies only to children who are critically ill within this setting. Table 4 outlines the suggestions provided by patient and family partners (third column), the decisions made by the clinical experts (fourth column), and resulting in the final scope elements following modifications (last column).

### Phase 2: Preparation

#### Step 3: Voting on Summary Recommendations and Prioritizing New Research Questions

##### Action 6: Vote on Existing Summary Recommendations

In July 2024, the clinical experts voted on 30 summary recommendations. All but 5 (26/30, 87%) achieved an agreement of >80%, with a 90% (19/21) response rate. The results of voting, consensus discussion, and patient and family partner validation are presented in Multimedia Appendix 9.

Following the consensus discussion, 23 transformed research questions were formulated from the 26 voted-upon summary recommendations as, during the consensus meeting, some summary recommendations were rejected, whereas others were merged or split into 2 distinct research questions.

##### Action 7: Prioritizing New Research Questions

**Figure 4:**
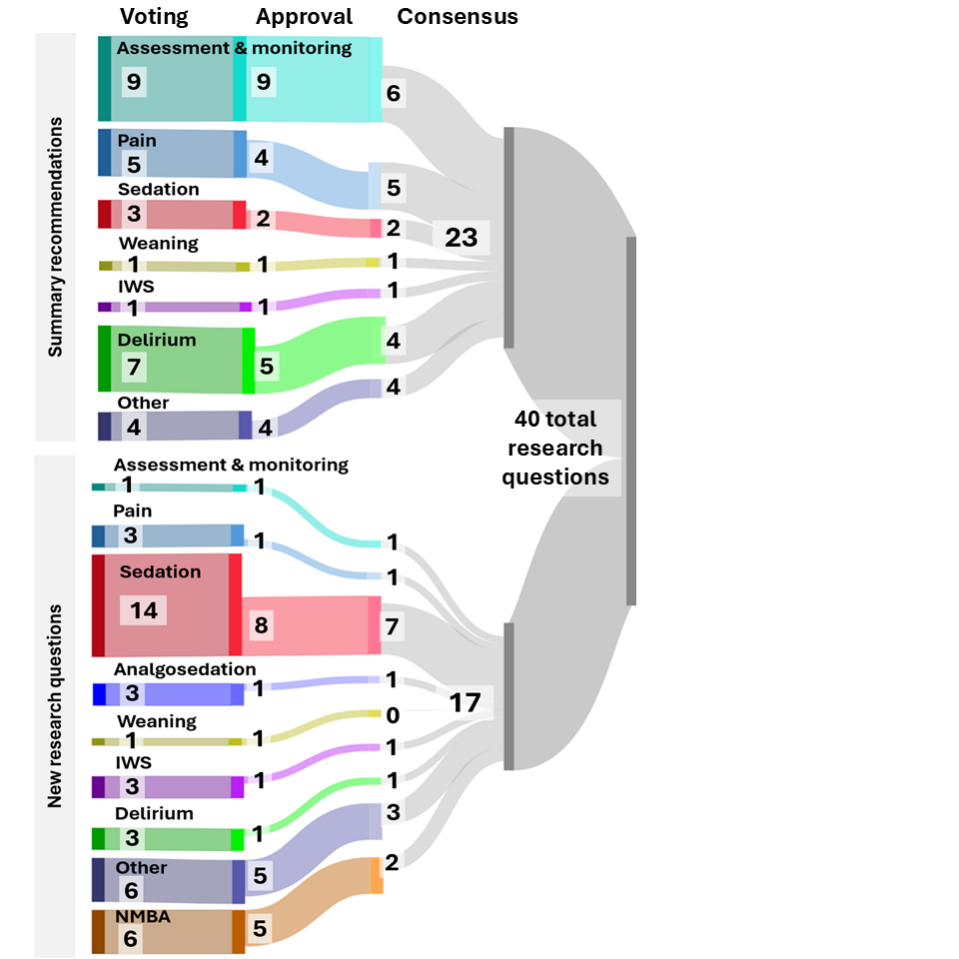
Clinical experts’ prioritization and consensus on summary recommendations and new research questions.

The clinical experts began the prioritization process of the new research questions in November 2024. A total of 52 new research questions were generated; the steering committee then consolidated these questions by removing duplicates or too specific questions into 40 (77%), which were put forth to the experts for prioritization using the 9-point Likert scale in the GRADEpro GDT. In total, 90% (19/21) of the clinical experts completed prioritization; of the 40 questions, 24 (60%) were rated as critically important (mean rating of 7-9), 16 (40%) were rated as important (mean rating of 4-6), and none were rated as not important (mean rating of 1-3). During a consensus meeting with 71% (15/21) of the panel members, the research questions were further reduced to 17 to be answered by the CPG (Multimedia Appendix 10 includes the new research question prioritization process with both clinical experts and patient and family partners and the consensus results). Figure 4 illustrates clinical experts’ prioritization and consensus across summary recommendations (action 6) and new research questions (action 7).

##### Action 8: Patient and Family Partner Input and Validation

In February 2025, the patient and family partners received their welcome email that included 3 SurveyMonkey links (one in English, one in French, and one in Italian based on the language preferences indicated in their expression of interest forms). Ten patient and family partners completed the survey: 7 (70%) in English, 3 (30%) in French, and none in Italian.

All patient and family partners agreed with the 23 transformed research questions resulting from the summary recommendations (ranging from 80% to 100%). These results can be found in Multimedia Appendix 9.

Of the 17 new research questions, using the median for determining the importance rating, 14 (82%) were rated as critically important, and 4 (24%) were rated as important (Multimedia Appendix 10). At the end of this section of the survey, patient and family partners were asked whether, from their perspective, there were any missing questions, and 6 were included from 40% (4/10) of the patient and family partners. However, these were not questions or were considered as included under the existing research questions (Multimedia Appendix 11 includes the suggested new research questions and the reasons for not including them).

The final list of all 40 research questions classified as either summary recommendations or new research questions is included in Multimedia Appendix 12 to clarify discrepancies as 3 summary recommendations were converted into new research questions but their classification varied between the two categories during the prioritization and voting processes.

#### Step 4: Identifying Source Guidelines and Matching New Research Questions With Recommendations

##### Action 9: Identifying Potential Source CPGs by Updating the Systematic Review of CPGs

**Figure 5:**
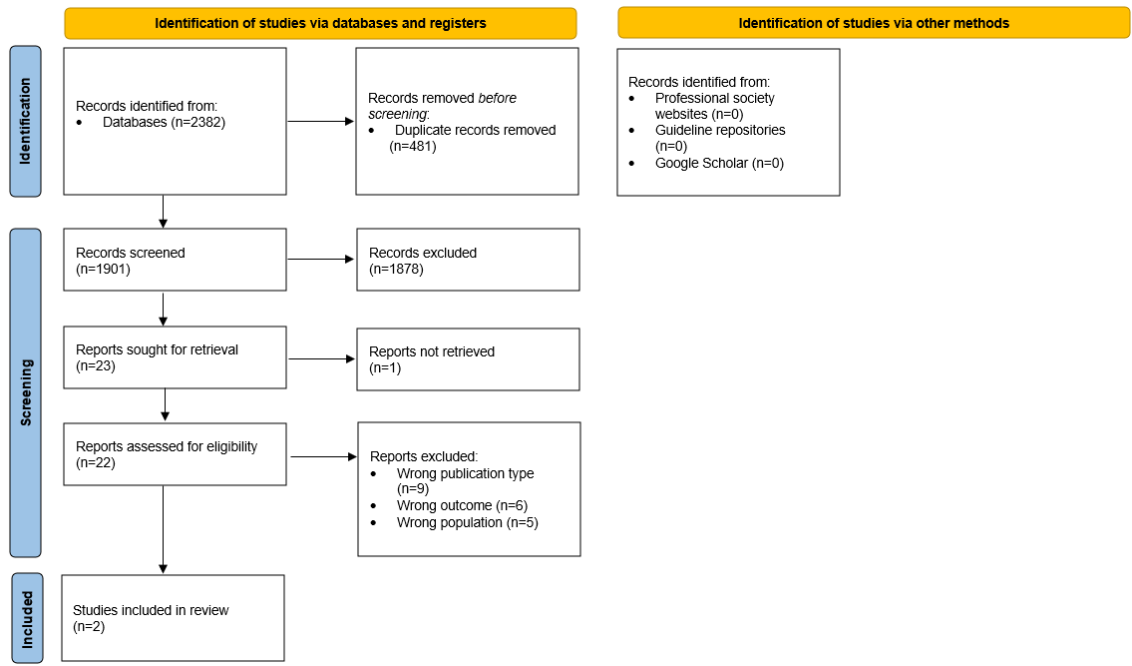
PRISMA (Preferred Reporting Items for Systematic Reviews and Meta-Analyses) flow diagram—updated search for the systematic review of clinical practice guidelines for managing pain, sedation, delirium, and iatrogenic withdrawal syndrome in pediatric intensive care units.

The searches were finalized in November 2024. A total of 1901 records were identified from the electronic databases after removing duplicates. Of these 1901 records, 22 (1.16%) studies underwent full-text review, from which 2 (9%) new CPGs were identified [80,81]. No additional CPGs were found through searches of guideline repositories, society websites, or Google Scholar. Figure 5 presents the search process as a PRISMA flow diagram [40]. Additional information on the excluded studies and the reasons for their exclusion can be found in Multimedia Appendix 13.

Both CPGs found were rated as low quality using the AGREE II instrument [34], therefore, will not be included in the next step of recommendation matching. The characteristics of the 2 included CPGs can be found in Multimedia Appendix 14, and the AGREE II consensus-based scores can be found in Multimedia Appendix 15.

##### Action 10: Matching New Research Questions With Existing Recommendations From Selected CPGs

Of the 17 new research questions, the steering committee agreed that 4 (24%) matched (see Multimedia Appendix 16 for details). Of these 4 questions, 3 (75%) were considered effectiveness questions and moved on to action 11.

#### Step 5: Health Outcome Prioritization (Action 11: Health Outcome Prioritization)

This process started in February 2025 with the clinical experts brainstorming health outcomes for each of the 3 effectiveness questions.

For the first question—“Should inhaled sedatives vs intravenous sedatives be used in difficult to sedate critically ill pediatric patients?”—the clinical experts prioritized 50% (16/32) of the health outcomes as critically important, whereas the remaining outcomes were rated as important. In contrast, the patient and family partners prioritized 92% (11/12) of the health outcomes as critically important, with the remaining outcome rated as important. Patient and family partners received a reduced list of health outcomes limited to those identified in the literature as the survey was sent at the same time as the clinical experts’ brainstorming process. However, they were given the opportunity to include any additional outcomes they considered important, effectively having a modified brainstorming process (Multimedia Appendix 17 presents the results of the health outcome prioritization for the 3 effectiveness research questions for both groups). By selecting the top 5 outcomes from each group, 7 health outcomes will be included in the evidence profile and EtD framework for this question.

For the second question—“Should sufentanil vs morphine be used for optimizing patient outcomes in mechanically ventilated critically ill children?”—the clinical experts prioritized 50% (18/36) of the health outcomes as critically important, whereas the remaining outcomes were rated as important.

For the third question—“Should fentanyl vs morphine be used for optimizing patient outcomes in mechanically ventilated critically ill children?”—the clinical experts prioritized 42% (14/33) of the health outcomes as critically important, whereas the remaining outcomes were rated as important.

For both the second and third questions, the patient and family partners prioritized 93% (13/14) of the health outcomes as critically important, with the remaining outcome rated as important. One patient and family partner added the health outcome of long-term consequences on cognitive development. By selecting the top 5 outcomes from each group, 9 health outcomes will be included in the evidence profile and EtD framework for these questions.

## Discussion

### Principal Findings

This protocol outlines a comprehensive methodology using the GRADE-ADOLOPMENT approach for adapting, developing, and contextualizing a CPG for managing pain, sedation, delirium, and IWS in children who are critically ill. By addressing existing gaps in clinical knowledge and practice, it will provide updated, evidence-based recommendations incorporating emerging research and clinical advancements. The rigorous methods included question and outcome prioritization. In phase 1 (setup), the steering committee established 3 groups: the steering committee, development panel, and patient and family partner advisory panel. A total of 21 clinical experts and 17 patient and family partners were recruited, representing diverse expertise, European countries, and lived experiences. Through structured voting and consensus processes, the scope of the CPG was finalized with 80% to 100% agreement among clinical experts and patient and family partners. In phase 2 (preparation), clinical experts voted on 30 summary recommendations compiled from existing medium- to high-quality CPGs, selecting 23 summary recommendations for inclusion. In addition, 17 new research questions were prioritized, bringing the total number of research questions to be addressed to 40. In total, 24% (4/17) of the new research questions matched existing recommendations. Using summary recommendations and matching to existing recommendations helps streamline the evidence review process. Of the 3 effectiveness questions, 1 (33%) had 7 prioritized health outcomes, whereas 2 (67%) had 9, all of which will be included in evidence profiles and EtD frameworks. The updated CPG search identified 2 low-quality CPGs, which were excluded from recommendation matching.

It is hypothesized that adhering to the GRADE and GRADE-ADOLOPMENT approaches for evaluating evidence quality and the strength of recommendations, along with the creation of evidence profiles and EtD frameworks (that will be readily available), will assist future CPG developers and updates. The methods used will ensure a high-quality, transparent, and accessible CPG that promotes consistent, standardized care and improved outcomes in PICUs. This protocol describes the GRADE-ADOLOPMENT approach when EtD tables are unavailable, offering a step-by-step process for those considering using this approach for CPG development or adaptation.

### Strengths and Limitations

A key strength of this CPG is the inclusion of patient and family partners early and throughout all phases of the CPG development process, addressing a limitation in previous CPGs [36]. This patient- and family-centered approach considers a broad range of patient and family partner experiences, enhancing the CPG’s relevance and applicability. In addition, involving a wide range of experts and patient and family partners from across Europe ensures representativeness and contextual sensitivity. The collaborative approach with continuous consensus meetings aims for alignment and buy-in from all development panel members. The summary recommendation voting, research question prioritization exercises, and identification of gaps in the literature as part of the recommendation table (action 18) will identify research gaps for prioritization and future research.

There are several limitations. First, achieving consensus may be challenging due to diverse perspectives across many stakeholders and languages. To establish consensus, a combination of surveys and meetings will be used to gather input. To address the language barriers, we will offer translated materials and captioning during web-based meetings. In addition, a bilingual speaker will always be present for direct translation when necessary. Another limitation inherent to the current state of knowledge is the availability and quality of evidence for certain conditions or interventions, which may be limited. However, by consolidating this information in a transparent and accessible manner and using surveys with the members of the large development panel will assist in forming credible recommendations. In addition, a standardized translation method for published studies might introduce new evidence previously not included.

### Comparison With Prior Work

Most societies have their own manuals outlining their processes for developing CPGs [82], but few have incorporated the GRADE-ADOLOPMENT approach. Until recently, there were limited published protocols specifically detailing CPG development [83-85] and none explicitly using the GRADE-ADOLOPMENT approach. Given the number of existing CPGs, the need for systematic adaptation instead of de novo development has led to a growing interest in the GRADE-ADOLOPMENT approach. However, the literature on its use primarily consists of broad methods included within published CPGs, leaving little room for detailed reporting, or postdevelopment publications presented as exemplars and discussions of experiences with the approach [86-88] rather than step-by-step protocols that can aid teams interested in this approach with writing their own protocol. To guide teams considering the GRADE-ADOLOPMENT approach, a recently published guidance document should be used to provide structure to their protocol and development process [19].

Despite advancement in methodological guidance, many society-level manuals fail to provide explicit direction on how to incorporate patient and family partners into the CPG development process [89]. Patient and family partner involvement has gained increasing interest as a means of ensuring that the recommendations meet patients’ needs. However, our review of existing CPGs for managing the 4 conditions found that almost none meaningfully included patient and family partners [36]. The methods for effectively integrating patient and family partners remain unclear, along with the definitions of how to describe their involvement and engagement [90]. Reporting is also limited to the postdevelopment phase [24,91]. When patient and family partners are involved, they most commonly participate in identifying key questions and reviewing the draft CPG [92], but their contributions often do not extend to formulating recommendations. This approach does not align with GRADE, which emphasizes the importance of considering benefits and harms to patients when formulating recommendations [18]. In this respect, patient and family partners have an important role in ensuring recommendations have the most impact on outcomes for future patients. Given the critical role that patient and family partners can play in shaping care strategies and improving health outcomes, their involvement should go beyond tokenistic participation to ensure that their perspectives are incorporated throughout the CPG development process in a transparently reported manner. Many groups are currently working to develop guidance on patient and family partner involvement, but these resources are not yet available [93,94] except for the newly published Guidelines International Network–McMaster Guideline Development Checklist extension for engagement [95].

### Conclusions

This protocol outlines a comprehensive approach to developing a CPG for managing pain, sedation, delirium, and IWS in pediatric patients who are critically ill that will integrate the most up-to-date evidence and diverse perspectives. By involving clinical experts and patient and family partners, the resulting CPG aims to be scientifically robust, patient centered, and contextually relevant.

Voting on summary recommendations and question prioritization, along with a thorough literature review to develop evidence profiles and EtD frameworks, ensures that the CPG addresses important clinical issues. This CPG is designed to promote optimal patient outcomes, making it valuable for clinicians, policy makers, patients, and families.

## Appendix

Multimedia Appendix 1 Checklist for guideline protocol reporting.

Multimedia Appendix 2 Declaration of interest form and conflict of interest—clinical experts.

Multimedia Appendix 3 Information sheet, expression of interest form including conflict of interest—patient and family partners.

Multimedia Appendix 4 Foundational data extraction—tracing and appraisal of guideline evidence, and study characteristics.

Multimedia Appendix 5 Evidence synthesis tables.

Multimedia Appendix 6 Recommendation drafting table.

Multimedia Appendix 7 Panel member role information and conflicts of interest.

Multimedia Appendix 8 Patient and family partner agreement with scope elements.

Multimedia Appendix 9 Evolution of voting on summary recommendations, consensus decision, and patient and family partner validation.

Multimedia Appendix 10 Evolution of voting on new research questions and consensus decision by clinical experts and patient and family partners.

Multimedia Appendix 11 Suggested new research questions by patient and family partners with reasons for exclusion.

Multimedia Appendix 12 List of 40 research questions to be answered by the guideline.

Multimedia Appendix 13 Excluded clinical practice guidelines with reasons.

Multimedia Appendix 14 Characteristics of 2 included clinical practice guidelines.

Multimedia Appendix 15 Appraisal of Guidelines for Research and Evaluation II consensus scores.

Multimedia Appendix 16 Decisions for recommendation matching of new research questions.

Multimedia Appendix 17 Top 5 health outcomes prioritized by each group.

## Abbreviations

AGREE: Appraisal of Guidelines for Research and Evaluation
COI: conflict of interest
CPG: clinical practice guideline
ESPNIC: European Society of Paediatric and Neonatal Intensive Care
EtD: evidence-to-decision
GDT: Guideline Development Tool
GRADE: Grading of Recommendations Assessment, Development, and Evaluation
IWS: iatrogenic withdrawal syndrome
PICU: pediatric intensive care unit
PRISMA: Preferred Reporting Items for Systematic Reviews and Meta-Analyses

## Data Availability

All data generated or analyzed during this study are included in this published article and its supplementary information files.
