# Corrigenda: Pain, Agitation, Delirium, and Iatrogenic Withdrawal Syndrome Management in Children Who Are Critically Ill: Protocol for a European Clinical Practice Guideline Using the Grading of Recommendations Assessment, Development, and Evaluation Approach

**DOI:** 10.2196/91949

**Published:** 2026-06-02

**Authors:** Ibo MacDonald, Alexia Cavin-Trombert, Cécile Jaques, Gwenaëlle De Clifford-Faugère, Pieter A De Cock, Saskia N de Wildt, Dmytro Dmytriiev, Juliane Engel, Paola Claudia Fazio, Sylvia George, Isabelle Goyer, Anna Harðardóttir, Julia Harris, Klára Horváth, Erwin Ista, Santiago Mencía, Tuuli Metsvaht, Maria Cristina Mondardini, Mehdi Oualha, Maria-Helena Perez, Krzysztof Pietrzkiewicz, Francesca Sperotto, Benjamin Wyness, Nilüfer Yalındağ, ESPNIC Nursing and Allied Healthcare Professionals Section, ESPNIC Pharmacology Section, Angela Amigoni, Anne-Sylvie Ramelet

**Affiliations:** 1 Institute of Higher Education and Research in Healthcare Faculty of Biology and Medicine University of Lausanne Lausanne Switzerland; 2 Medical Library Lausanne University Hospital and University of Lausanne Lausanne Switzerland; 3 Department of Pharmacy Ghent University Hospital Ghent Belgium; 4 Department of Basic and Applied Health Sciences Ghent University Ghent Belgium; 5 Department of Pediatric Intensive Care Ghent University Hospital Ghent Belgium; 6 Department of Pharmacy, Pharmacology and Toxicology Radboud University Medical Center Nijmegen The Netherlands; 7 Department of Neonatal and Pediatric Intensive Care - Pediatric Intensive Care Erasmus MC – Sophia Children’s Hospital Erasmus University Medical Center Rotterdam The Netherlands; 8 Department of Anesthesiology and Intensive Care Vinnitsya National Medical University Vinnitsya Ukraine; 9 Intensive Care Medicine and Pulmonology Department of Cardiology University Children's Hospital Tuebingen Tuebingen Germany; 10 Pediatric Intensive Care Unit University-Hospital of Padua Padua Italy; 11 Paediatric Critical Care Unit & Department of Pharmacy Oxford University NHS Foundation Trust Oxford United Kingdom; 12 Department of Pharmacy, Pediatrics, Anaesthesia and Critical Care University Hospital of Caen Caen France; 13 Intensive Care Unit National University Hospital of Iceland Reykjavík Iceland; 14 Division of Children’s Nursing London South Bank University London United Kingdom; 15 Pediatric Center Semmelweis University Budapest Hungary; 16 Section Nursing Science Department of Internal Medicine, Erasmus MC Erasmus University Medical Center Rotterdam The Netherlands; 17 Pediatric Intensive Care Unit, Gregorio Marañón Hospital Complutense University of Madrid Madrid Spain; 18 Institute of Clinical Medicine, Department of Paediatrics University of Tartu Tartu Estonia; 19 Paediatric and Neonatal Intensive Care Unit Tartu University Hospital Tartu Estonia; 20 Pediatric Anesthesia and Intensive Care Unit, Department of Woman's and Child's Health IRCCS AOUBO Bologna Italy; 21 Pediatric Intensive Care Unit Necker University Hospital Paris France; 22 Paris City University Paris France; 23 Paediatric Intensive and Intermediate Care Units, Service of Pediatrics, Women-Mother-Child Department Lausanne University Hospital Lausanne Switzerland; 24 Faculty of Biology and Medicine University of Lausanne Lausanne Switzerland; 25 Department of Paediatric Anaesthesiology and Intensive Therapy Poznan University of Medical Sciences Poznan Poland; 26 Department of Cardiology Boston Children's Hospital Boston, MA United States; 27 Department of Pediatrics Harvard Medical School Boston, MA United States; 28 Pharmacy Department Cambridge University Hospitals NHS Foundation Trust Cambridge United Kingdom; 29 School of Medicine Marmara University Istanbul Turkey; 30 Division of Pediatric Critical Care Department of Pediatrics Marmara University Pendik Training and Research Hospital Istanbul Turkey; 31 See Acknowledgements; 32 Pediatric Intensive Care unit University Hospital of Padova Padova Italy

In “Pain, Agitation, Delirium, and Iatrogenic Withdrawal Syndrome Management in Children Who Are Critically Ill: Protocol for a European Clinical Practice Guideline Using the Grading of Recommendations Assessment, Development, and Evaluation Approach”, the authors added two groups to the author list. The following is the corrected list of authors:

Ibo MacDonald, Alexia Cavin-Trombert, Cécile Jaques, Gwenaëlle De Clifford-Faugère, Pieter A De Cock, Saskia N de Wildt, Dmytro Dmytriiev, Juliane Engel, Paola Claudia Fazio, Sylvia George, Isabelle Goyer, Anna Harðardóttir, Julia Harris, Klára Horváth, Erwin Ista, Santiago Mencía, Tuuli Metsvaht, Maria Cristina Mondardini, Mehdi Oualha, Maria-Helena Perez, Krzysztof Pietrzkiewicz, Francesca Sperotto, Benjamin Wyness, Nilüfer Yalındağ, ESPNIC Nursing and Allied Healthcare Professionals Section, ESPNIC Pharmacology Section, Angela Amigoni, Anne-Sylvie Ramelet

The correction will appear in the online version of the paper on the JMIR Publications website, together with the publication of this correction notice. Because this was made after submission to PubMed, PubMed Central, and other full-text repositories, the corrected article has also been resubmitted to those repositories.

