# Supplementary material for: Pain, Agitation, Delirium, and Iatrogenic Withdrawal Syndrome Management in Children Who Are Critically Ill: Protocol for a European Clinical Practice Guideline Using the Grading of Recommendations Assessment, Development, and Evaluation Approach
Source: JMIR Res Protoc. 2025 Sep 8;14:e67930. doi: 10.2196/67930 (PMC12455155; doi:10.2196/67930)
Supplement: Multimedia Appendix 1 [file resprot_v14i1e67930_app1.pdf]

SUPPORTING INFORMATION S1 – Checklist for guideline protocol

**Protocol element**	**Included in protocol**	**Location: page**
Guideline registration	Pre-registered with PREPARE	Methods – page 3
Guideline development group<br>1) Experts and patient groups	Yes	Methods – phase 1: step 1 - 3 – pages 3 - 7
2) Planned composition and responsibilities of the panel	Yes	Methods – pages 6-7 + Table 1.
3) Names and titles of each member	Multimedia appendices S7	
Conflict of interest management<br>1) Forms	Yes	Methods – page 3 [experts] and Multimedia appendix 2; pages 4 [patient and family partners]. Multimedia appendix 3
2) How COI would be managed	Yes	Methods – page 7
Determining of clinical questions and outcomes	Yes + summary recommendations	Methods – pages 8-10
Evidence retrieval and assessment<br>1) Databases	Yes	Page 13
2) Types of included studies	Yes	Page 13 + Figure 3
3) Appraisal of studies	Yes	Pages 14
4) Rating system for evidence and recommendations	Yes	Evidence quality : page 15<br>Recommendations: page 16-17
5) Details on how the systematic review will be conducted	Yes	Pages 12-13
Methods used to reach consensus	Yes	-Scope/population of guideline: page 8<br>-Voting summary recommendations: page 8-9<br>-Research question prioritization: page 9-10<br>-Confirmation of summary recommendation and new research questions by patients and families: pages 10<br>-Outcome prioritization: page 11-12<br>-Study selection: page 13<br>-Recommendation drafting and voting: page 17<br>-Final guideline approval: page 19
Other:<br>1) External review	Yes	Pages 19
2) Plans for guideline implementation and dissemination	Dissemination only	Dissemination: page 19
3) Updating	Yes	Page 19
4) Flow chart of development steps and processes	Yes	Figure 1 and Figure 2
5) Timeline for development	Yes	Figure 1
