# Supplementary material for: Pain, Agitation, Delirium, and Iatrogenic Withdrawal Syndrome Management in Children Who Are Critically Ill: Protocol for a European Clinical Practice Guideline Using the Grading of Recommendations Assessment, Development, and Evaluation Approach
Source: JMIR Res Protoc. 2025 Sep 8;14:e67930. doi: 10.2196/67930 (PMC12455155; doi:10.2196/67930)
Supplement: Multimedia Appendix 2 [file resprot_v14i1e67930_app2.pdf]

SUPPORTING INFORMATION S2 – Declaration of interest form and conflict of interest: Expert panel

If you wish to become a member of the GDG you must consider whether or not you have any interests that could conflict with your role. Conflicts of interest have to be declared when you are appointed and then every year. All GDG members must declare conflicts of interest before starting work on a guideline.
The information you provide on this form will be used to assess if you have any potential conflicts of interest, we ask for this information to comply with our organisational policies.

**Name:**
**Role/Profession:**
**E-mail:**
**Tel:**
**Date:**
**Signature:**

Have you personally received any financial reimbursement from any drug/technology companies?
Personal specific interest if you have worked within the last two years on a project for the healthcare industry and have personally received payment for that work in any form or any non-financial interest. If the interest is no longer current, you may declare it as a lapsed personal specific interest.

- **Yes** ☐
- **No** ☐

If you have received any financial reimbursement, please state from which company (please list all if more than one) and the name of the drug/technology (if appropriate). *
Personal non-specific interest if you have a current personal non-specific interest in the healthcare industry concern which does not relate specifically to the guideline area under consideration.

If you have received any financial reimbursement towards your department, please state from which company (please list all if more than one) and also the name of the Drug/technology (if appropriate).
Non-personal specific interest if you are aware that the department for which you are responsible has, in the last five years, been working in the area of the guideline, but you have yet to personally receive payment in any form from the healthcare industry for the work done.

If the department you are employed by has received any financial reimbursement, please state from which company (please list all if more than one) and also the name of the drug/technology (if appropriate).

Non-personal, non-specific interest if you are aware that the department for which you are responsible is currently receiving payment from the healthcare industry concerned, which does not relate specifically to the product under consideration.

If any of your family members received any financial reimbursement, please state from which company (please list all if more than one) and also the name of the drug/technology (if appropriate).

Family interest. In the last 12 months, has a member of your family had any financial involvement with the healthcare industry, or are they planning to have such financial involvement? This could include: holding a directorship or other paid position or carrying out consultancy or fee-paid work, or having shareholdings or other beneficial interests, or receiving expenses and hospitality over and above what would be reasonably expected to attend meetings and conferences.

Are the above interests specific to the product or services being evaluated for the named guideline topic?

- **Yes ☐**
- **No ☐**

Other additional relevant information eg. membership with other organisations

Confirmation

I confirm that the information provided above is complete and correct. I acknowledge that any changes in these declarations during my work with the GDG must be notified to the guideline steering committee as soon as practicable and no later than 28 days after the interest arises. I am aware that if I do not make complete, accurate and timely declarations, this may result in being removed from the GDG if there has been a deliberate breach of the declaration policy.

I declare that the above details are correct to the best of my knowledge on ☐

Signed : ________________________________ Date: ____________________________
