# Supplementary material for: Pain, Agitation, Delirium, and Iatrogenic Withdrawal Syndrome Management in Children Who Are Critically Ill: Protocol for a European Clinical Practice Guideline Using the Grading of Recommendations Assessment, Development, and Evaluation Approach
Source: JMIR Res Protoc. 2025 Sep 8;14:e67930. doi: 10.2196/67930 (PMC12455155; doi:10.2196/67930)
Supplement: Multimedia Appendix 3 [file resprot_v14i1e67930_app3.pdf]

SUPPORTING INFORMATION S3 – Information sheet: Patient and family partners

Invitation to partner in Guideline Development for the management of pain and comfort in critically ill children

Dear Patient or Family Partners,

We would like to invite you to become involved as a valued member of our guideline development panel or guideline advisory panel.

Guidelines are made by summarizing the best available evidence, providing healthcare professionals with advice on the best care and treatment for certain patient groups or conditions.

We are a group of healthcare professionals who, as part of the European Society of Paediatric and Neonatal Intensive Care, are developing a European guideline for managing pain, sedation, delirium, and withdrawal in the pediatric intensive care unit (PICU) for healthcare professionals. It is very important to us that the development of this guideline is carried out in a patient-family friendly and oriented way. Therefore, we need to include your perspectives, insights, and experiences.

Who is on the guideline development panel?
The panel is made up of healthcare professionals, including doctors, nurses, and pharmacists, alongside patient and family partners, all of whom contribute equally.

Why you?
As a parent or child who has experienced hospitalization in the PICU, you bring unique and invaluable insights and expertise into the impact of hospitalization on life, work/school, and family. By sharing your perspective, you can make a huge difference in how healthcare is delivered in the future, ensuring that the guideline is comprehensive, patient-centered, and responsive to the needs of patients and their families in the PICU.

What knowledge and experience do you need?
We are looking for people who have firsthand experience with the PICU environment, especially those who experienced or had a child undergo mechanical ventilation for 3 days or more.

As a member of the guideline development panel:	*As a member of the guideline advisory panel*:
• You must be able to understand, read and communicate in English.	• For the question and outcomes voting, there are no language requirements, as these can be translated if needed.<br>• You do need to be able to read English for reviewing the draft recommendations and guidelines, but you can provide feedback in your own language.

Description of involvement in this opportunity

There are two ways to participate in the guideline development process: 1) as a member of the guideline development panel, or 2) as a member of the guideline advisory panel. Below, we have outlined the differences between the two roles using two columns.

As a member of the guideline development panel:<br><br>• You will attend 4-5 guideline development panel meetings, held on-line using zoom.<br>• You will take part in voting on research questions and outcomes.<br>• You will participate in discussions that will shape the guideline and represent the views and experiences of patients and families to ensure these are considered by the guideline development panel. Most importantly, you will provide input on the benefits, harms, and values relevant to patients and families.<br>• You will participate in recommendation development.<br>• You will review and comment on documents between meetings.<br>• You will review the near-final recommendations and guideline.<br><br>The timeline of your commitment spans from June 2024 to February 2025. (with a break over the summer).<br><br>The time commitment, including preparation and attendance at meetings, is estimated to be 8-9 hours in total.<br><br>You will receive the opportunity to have mentoring discussions before and after each meeting.	*As a member of the guideline advisory panel*:<br><br>• You will be asked to participate in voting on research questions and prioritizing outcomes. These can be translated to your local language.<br>• You will also be asked to review the near-final recommendations and guideline, but these will be in English.<br><br>All input will be done using surveys or email.<br><br>The timeline of your commitment spans from June 2024 to February 2025.<br><br>Time commitment for the voting, prioritizing, and reviewing documents, is estimated to be 3-4 hours in total.

All patient and family partners will be given a list of resources for on-line training on guideline development.

We assume that everyone involved will do their best to make this collaboration work. However, in case of unforeseen circumstances, you may stop your involvement as a patient and family partner at any time by notifying your contact person. You do not need to specify any reason.

You may be asked to participate in the development of easy-to-understand materials for parents if this need is identified when formulating recommendations. We may contact you to ask for your support.

What's in it for me?

- You will be helping to develop a European guideline on managing pain, sedation, delirium, and withdrawal to make care and treatment better for children in the PICU.
- As a valued partner of the guideline development panel, you are the expert by experience and an equal contributor alongside the healthcare professionals, you will help to bring your understanding of the experience to light for the rest of the team.
- You will receive training and experience in guideline development.
- Your name will be included in all publications (if you choose to have it included).

How will I be compensated?

As a member of the guideline development panel:<br><br>• You will receive an hourly compensation of 60€ for guideline development panel meetings.<br>• You will be paid a flat rate of 90€ for each task completed outside of panel meetings, including: 1) voting on question, 2) outcome prioritization, 3) recommendation review, and 4) draft guideline review.	*As a member of the guideline advisory panel*:<br><br>You will be paid a flat rate of 90€ for each advisory panel task completed: 1) voting on question, 2) outcome prioritization, 3) recommendation review, and 4) draft guideline review.

What happens after I apply?

As a member of the guideline development panel:<br><br>• You will receive all information to participate<br>• Your work will be acknowledged in future publications.	*As a member of the guideline advisory panel*:<br><br>• All interested advisory panel members will be incorporated into the guideline development process.<br>• You will receive all information to participate<br>• Your work will be acknowledged in future publications

For more information, please contact:
(Each expert development panel member to add their contact information here)
To express your interest in participating please complete the following form.

Expression of interest form: Patient and family partners

Patient and Family Partner expression of interest in Guideline Development for the management of pain and comfort in critically ill children

Thank you for your interest in becoming a Patient and Family Partner in the development of our ESPNIC guideline for managing pain, sedation, delirium, and iatrogenic withdrawal in the paediatric intensive care unit (PICU).

Patient and family partners are former patients and carers of loved ones who have had experience with the PICU. Patient and family partners join either the development panel or the advisory panel to share their experiences and perspectives.

In this form we ask for the following personal information from you:

- **A.** Which partnership opportunity are you interested in: a) guideline development or b) guideline advisory panel.
- **B.** Contact information: your name, address, phone number, email, preferred language for communication, and banking information.

For those expressing interest in the Guideline development panel, we also ask for:

- **C.** Information about your experience with the PICU: type of treatment, length of PICU stay, purpose of admission.
- **D.** The completion of conflict of interest.

We collect this information to establish contact lists of patient and family partners for both guideline development opportunities. If we receive too many expressions of interest for the guideline development panel, we will use the PICU experience information to select the most diverse members. This information will not be shared with anyone except the guideline steering committee for selection purposes, if needed.

If you have questions, please email: (insert contact information of expert panel member)

Partnership opportunity selection agreement

The ESPNIC Guideline develop steering committee and expert panel members want our Patient and Family Partners to have a positive experience working with us on the development of this important guideline. Having this agreement about your rights and responsibilities will help you have safe, high quality, and meaningful engagements. As a Patient or Family Partner:

You have the right to:

- Feel safe.
- Be treated with respect, dignity, and fairness.
- Have your privacy and confidentiality protected.
- Be provided with information and support so you can take part in engagements.
- Be recognized and compensated for your time.
- Know the outcome of engagements you take part in.
- Leave engagement at any time.

You are expected to:

- Treat people with respect, dignity, and fairness.
- Participate in the engagement activities, meet your roles and responsibilities as best as you can.
- Respect the privacy of others and not share any personal information about others.
- Not share any confidential information about engagement.

I am interested in collaborating on:

__ I wish to participate on the Guideline development panel

__ I wish to participate on the Guideline advisory panel

A. Contact information

By providing your name and contact details, I confirm my agreement with the rights and expectation outlined on the previous page.

Parent

First name:________________________________ Last name:_____________________________

Email:____________________________________ Phone number:_________________________

Address:____
____

Postal code:______________________________ Country of residence:_________________

Patient (for patients that cannot consent, parents must also partner)

First name:________________________________ Last name:______________________________

Email:____________________________________ Phone number:__________________________

Age______________________

Preferred language for question and outcome voting, if not English:______________________

Banking account details

Full name of account holder ____

Name of bank ____

Bank address ____

IBAN ____

BIC/SWIFT code ____

B. Information on PICU experience

Please complete the table with the details about your or your child's PICU experience

Reason for PICU admission	
Number of days admitted	
Number of admissions	
Length of mechanical ventilation (hours)	
Gender	
Age (D.O.B: dd.mm.year)	
Time since last admission (years, months)	
Did you or your child experience delirium?	
Did you or your child experience iatrogenic withdrawal syndrome?	

C. Conflict of interest

All guideline development panel members are asked to declare any potential conflict of interest, or possible competing interest.

What does this mean?
A conflict of interest or a competing interest is an interest which may influence your input and decisions when discussing guideline content.
Therefore, we are asking you to declare any financial or non-financial conflicts and interests you or your partner, close relatives, may be involved in.
The information you provide in this part of the expression of interest form will be used by the Guideline Steering Committee to see if you have any potential conflicts of interest. All panel members' conflicts of interest will be disclosed in all publications related to the guideline. This is an essential step for establishing trust in the content of the guideline.

Do you have any financial interests that needs to be declared, for either you, your partner or a close relative? Specifically related to the healthcare industry. This question is related to anything of monetary value, including salary and payments for services; equity interests (like stocks or stock options); and intellectual property rights (patents and copyrights and royalties arising from such interests).

☐ Yes ☐ No

If you answered yes, please provide details:

Do you have any non-financial professional or personal interest that needs to be declared? This could include membership in a healthcare advocacy group, member in a professional society, participation in another research project aiming to determine the effectiveness of an intervention that might become something the guideline considers.

☐ Yes ☐ No

If you answered yes, please provide details:

Confirmation

I confirm that the information provided above is complete and correct. I acknowledge that any changes to my conflicts of interest during my work with the Guideline development panel will be made to the guideline steering committee as soon as possible. I am aware that if I do not make complete, accurate and timely declarations, this may result in being removed from the guideline development panel if there has been a deliberate breach of the declaration policy.

I declare that the above details are correct to the best of my knowledge:

Name ________________________________ Date: ________________________

Signature:
