# Supplementary material for: Pain, Agitation, Delirium, and Iatrogenic Withdrawal Syndrome Management in Children Who Are Critically Ill: Protocol for a European Clinical Practice Guideline Using the Grading of Recommendations Assessment, Development, and Evaluation Approach
Source: JMIR Res Protoc. 2025 Sep 8;14:e67930. doi: 10.2196/67930 (PMC12455155; doi:10.2196/67930)
Supplement: Multimedia Appendix 4 [file resprot_v14i1e67930_app4.pdf]

Initial data extraction tables

Summary recommendation: [insert text]

Transformed research question: [insert text]

Table 1: Referenced studies from each recommendation used to create the summary recommendation

Guideline	Recommendation, body of guideline	References	Relevance to rec.	Level of support to rec
			1 = very, 2 = somewhat, 3 = a little, 4 = not at all	

Characteristics of included studies

Table 2: Study characteristics

Study reference	Type of study	Setting	Population		Intervention description	Outcomes (primary (P), secondary (S)	Results	Key conclusions	Patient and family perspectives	Assessment of study quality
			Intervention (I)	Control (C)						
			[Comparative studies]							
			[non-comparative studies, narrative]							
