# Supplementary material for: Pain, Agitation, Delirium, and Iatrogenic Withdrawal Syndrome Management in Children Who Are Critically Ill: Protocol for a European Clinical Practice Guideline Using the Grading of Recommendations Assessment, Development, and Evaluation Approach
Source: JMIR Res Protoc. 2025 Sep 8;14:e67930. doi: 10.2196/67930 (PMC12455155; doi:10.2196/67930)
Supplement: Multimedia Appendix 5 [file resprot_v14i1e67930_app5.pdf]

Evidence synthesis tables

Table 1: Evidence profile (grouped by outcome)

Certainty assessment							Summary of findings					Importance	Comments
No. of studies	Design	Risk of bias	Inconsistency	Indirectness	Imprecision	Other considerations	No of patients		Effect		Certainty		
							Interventions	Usual care	Relative	Absolute			
Outcome#1													
Outcome#2													

Table 2: Evidence-to-decision framework

Criteria	Judgements	Research evidence	Comments
**Problem:**<br><br>**Is the problem a priority?**	○ No<br>○ Probably no<br>○ Uncertain<br>○ Probably yes<br>○ Yes<br>○ Varies		
**Certainty of evidence:**<br><br>**What is the overall certainty of this evidence?** (consider desirable and undesirable effects)	○ No included studies<br>○ Very low<br>○ Low<br>○ Moderate<br>○ High	**EtD table(s) go here.**	

Criteria	Judgements	Research evidence	Comments
**Are the desirable anticipated effects large?**	○ No<br>○ Probably no<br>○ Uncertain<br>○ Probably yes<br>○ Yes<br>○ Varies		
**Are the undesirable anticipated effects small?**	○ No<br>○ Probably no<br>○ Uncertain<br>○ Probably yes<br>○ Yes<br>○ Varies		
**Value:**<br><br>**Is there important uncertainty about how much people value the main outcomes?**	○ Important uncertainty or variability<br>○ Possibly important uncertainty or variability<br>○ Probably no important uncertainty of variability<br>○ No important uncertainty of variability<br>○ No known undesirable		
**Balanced effect:**<br><br>**Are the desirable effects large relative to undesirable effects?**	○ No<br>○ Probably no<br>○ Uncertain<br>○ Probably yes<br>○ Yes<br>○ Varies	(based on previous category answers)	

Criteria	Judgements	Research evidence	Comments
**Resources:**<br>**Are the resources required small?**<br>**Is the incremental cost small relative to the net benefits?**	○ No<br>○ Probably no<br>○ Uncertain<br>○ Probably yes<br>○ Yes<br>○ Varies		
**Equity:**<br>**What would be the impact on health inequities?**	○ Increased<br>○ Probably increased<br>○ Uncertain<br>○ Probably reduced<br>○ Reduced<br>○ Varies		
**Acceptability:**<br>**Is the option acceptable to key stakeholders?**	○ No<br>○ Probably no<br>○ Uncertain<br>○ Probably yes<br>○ Yes<br>○ Varies		
**Feasibility**<br>**Is the option feasible to implement?**	○ No<br>○ Probably no<br>○ Uncertain<br>○ Probably yes<br>○ Yes<br>○ Varies		

Adapted from Alonso-Coello P, Schunemann HJ, Moberg J, Brignardello-Petersen R, Akl EA, Davoli M, et al. GRADE Evidence to Decision (EtD) frameworks: a systematic and transparent approach to making well informed healthcare choices. 1: Introduction. BMJ. 2016;353:i2016.10.1136/bmj.i2016

Tables 3: Summary judgments

Summary judgement table – based on GRADEpro GDT [1]

<table>
<tr><td>Criteria</td><td colspan="8">Summary of judgements</td></tr>
<tr><td>Problem</td><td>No</td><td colspan="2">Probably no</td><td colspan="2">Probably yes</td><td>Yes</td><td>Varies</td><td>Don’t know</td></tr>
<tr><td>Certainty of evidence</td><td>Very low</td><td colspan="2">Low</td><td colspan="2">Moderate</td><td>High</td><td colspan="2">No included studies</td></tr>
<tr><td>Desirable effect</td><td>Trivial</td><td colspan="2">Small</td><td colspan="2">Moderate</td><td>Large</td><td>Varies</td><td>Don’t know</td></tr>
<tr><td>Undesirable effect</td><td>Trivial</td><td colspan="2">Small</td><td colspan="2">Moderate</td><td>Large</td><td>Varies</td><td>Don’t know</td></tr>
<tr><td>Values</td><td>Important uncertainty or variability</td><td colspan="2">Possibly important uncertainty or variability</td><td colspan="2">Probably no important uncertainty or variability</td><td>No important uncertainty or variability</td><td colspan="2"></td></tr>
<tr><td>Balance of effect</td><td>Favors the comparison</td><td>Probably favors the comparison</td><td colspan="2">Neither</td><td>Probably favors the interventions</td><td>Favors the intervention</td><td>Varies</td><td>Don’t know</td></tr>
<tr><td>Resources required</td><td>Large costs</td><td>Moderate costs</td><td colspan="2">Negligible costs and saving</td><td>Moderate savings</td><td>Large savings</td><td>Varies</td><td>Don’t know</td></tr>
<tr><td>Equity</td><td>Reduced</td><td>Probably reduced</td><td colspan="2">Probably no impact</td><td>Probably increase</td><td>Increased</td><td>Varies</td><td>Don’t know</td></tr>
<tr><td>Acceptability</td><td>No</td><td colspan="2">Probably no</td><td colspan="2">Probably yes</td><td>Yes</td><td>Varies</td><td>Don’t know</td></tr>
<tr><td>Feasibility</td><td>No</td><td colspan="2">Probably no</td><td colspan="2">Probably yes</td><td>Yes</td><td>Varies</td><td>Don’t know</td></tr>
</table>

1. GRADEpro GDT. GRADEpro Guideline Development Tool. 2020
