# Supplementary material for: Pain, Agitation, Delirium, and Iatrogenic Withdrawal Syndrome Management in Children Who Are Critically Ill: Protocol for a European Clinical Practice Guideline Using the Grading of Recommendations Assessment, Development, and Evaluation Approach
Source: JMIR Res Protoc. 2025 Sep 8;14:e67930. doi: 10.2196/67930 (PMC12455155; doi:10.2196/67930)
Supplement: Multimedia Appendix 6 [file resprot_v14i1e67930_app6.pdf]

Recommendation table – based on GRADEpro GDT [1]

**Type of recommendation**	Strong recommendation against the intervention	Conditional recommendation against the intervention	Conditional recommendation for either the intervention or the comparison	Conditional recommendation for the intervention	Strong recommendation for this intervention	Good practice statement
**Recommendation**	Insert wording for the recommendation					
**Justification**	Consider: health benefits, harms, patient preferences, feasibility (including costs)					
**Subgroup considerations**	Explanation of different subgroups who may have different outcomes or need different approaches					
**Implementation considerations**	Barriers and facilitators to implementation					
**Monitoring and evaluation**	Criteria for monitoring and evaluating the impact of the recommendation when implemented.					
**Research priorities**	Areas where research evidence was not identified					
**Suggestions for accompanying materials**	Consider – resources to help with implementing the recommendation, educational materials, monitoring criteria. These could be established resources (add references or links) or resources that needs to be created					

1. GRADEpro GDT. GRADEpro Guideline Development Tool. 2020
