# Supplementary material for: Pain, Agitation, Delirium, and Iatrogenic Withdrawal Syndrome Management in Children Who Are Critically Ill: Protocol for a European Clinical Practice Guideline Using the Grading of Recommendations Assessment, Development, and Evaluation Approach
Source: JMIR Res Protoc. 2025 Sep 8;14:e67930. doi: 10.2196/67930 (PMC12455155; doi:10.2196/67930)
Supplement: Multimedia Appendix 7 [file resprot_v14i1e67930_app7.pdf]

Name	Panel	Profession	Organization	Country	Content expertise	Role	Conflict of interest
Ibo MacDonald	Steering committee - lead	Nurse	University of Lausanne	Switzerland	Pain, Sedation, Delirium, Withdrawal, Psychometrics, Systematic review and meta-analysis - methodology, GRADE – methodologist, Nursing practice and research	Methodological expert	None
Angela Amigoni	Steering committee – co-lead	Doctor	University Hospital of Padova	Italy	Pain, Sedation, Delirium, Withdrawal, Practice and research for physicians	Physician expert	None
Anne-Sylvie Ramelet	Steering committee – co-lead	Nurse	University of Lausanne	Switzerland	Pain, Sedation, Delirium, Withdrawal, Psychometrics, Systematic review - methodology, Nursing practice and research	Nurse expert	None
Alexia Cavin-Trombert,	Invited member	Health Sciences librarian	Lausanne University Hospital and University of Lausanne	Switzerland	Library science	Health Sciences librarian	None
Cécile Jaques	Invited member	Health Sciences librarian	Lausanne University Hospital and University of Lausanne	Switzerland	Library science	Health Sciences librarian	None
Gwenaëlle De Clifford-Faugère	Development panel	Nurse	University of Lausanne	Switzerland	Pain, Nursing practice, Psychometric	Clinical expert – screening, appraisal, data	None

**Name**	**Panel**	**Profession**	**Organization**	**Country**	**Content expertise**	**Role**	**Conflict of interest**
					properties, Systematic review and meta-analysis - methodology	extraction, guideline recommendation development, writing, and consensus participation	
Pieter A. De Cock	Development panel	Pharmacist-clinical pharmacologist	Ghent Hospital University and Ghent University	Belgium	Pharmacology related to pain, sedation, delirium and IWS in PICU		None
Saskia N. de Wildt	Development panel	Doctor	Radboud University Medical Center	The Netherlands	Pharmacology, Sedation		Not related to current protocol: Dr de Wildt is DSMC chair for Khondrion, consultancy fee reimbursed to employer
Dmytro Dmytriiev	Development panel	Doctor	Vinnitsya National Medical University	Ukraine	Pain, Pediatric anesthesiology		None
Juliane Engel	Development panel	Doctor	University Children's Hospital	Germany	Pediatric Intensive Care Medicine, Longterm Outcome PICS, Analgesia, Sedation and Delirium Management		None

Name	Panel	Profession	Organization	Country	Content expertise	Role	Conflict of interest
Paola Claudia Fazio	Development panel	Nurse	University-Hospital of Padua	Italy	Critical Care, Pain assessment and management, Sedation practices, Abstinence and delirium prevention and treatment, Enteral feeding protocols, PICU		None
Sylvia George	Development panel	Pharmacist	Oxford University NHS Foundation trust	UK	Pharmacokinetics, Pharmacodynamics, pediatric critical care pharmacotherapy, analgo-sedation and withdrawal management		None
Isabelle Goyer	Development panel	Pharmacist	University Hospital of Caen	France	Pharmacotherapy, Clinicial pharmacology, specifically drug management of the critically ill children		None
Anna Harðardóttir	Development panel	Nurse	Landspitali, The National University Hospital in Reykjavík	Iceland	Pediatric intensive care nursing		None
Julia Harris	Development panel	Nuse	London South Bank University	UK	Pain and Sedation assessment and management, PICU		None

Name	Panel	Profession	Organization	Country	Content expertise	Role	Conflict of interest
Klára Horváth	Development panel	Doctor	Semmelweis University	Hungary	Sleep, Circadian rhythm, Sedation		None
Erwin Ista	Development panel	Nurse	Erasmus MC – Sophia Children's Hospital	The Netherlands	Pain, Sedation, Delirium, Withdrawal, Psychometrics, PICU, Early mobilization, Nursing practice and research		None
Santiago Mencía	Development panel	Doctor	Gregorio Marañón Hospital	Spain	Analgosedation		None
Tuuli Metsvaht	Development panel	Doctor	University of Tartu	Estonia	Pharmacokinetics, Pharmacodynamics, Sedation, PICU, NICU, Clinical research		None
Maria Cristina Mondardini	Development panel	Doctor	IRCCS AOUBO, Bologna	Italy	Pediatric pain management, Analgesia and sedation in PICU, Pediatric palliative care		None
Mehdi Oualha	Development panel	Doctor	Necker University Hospital	France	Pediatric critical care, Pediatric clinical pharmacology		None
Maria-Helena Perez	Development panel	Doctor	University Hospital and University of Lausanne	Switzerland	PICU, Hemodynamics, Pain, Sedation, Withdrawal, Delirium, Quality improvement		None

Name	Panel	Profession	Organization	Country	Content expertise	Role	Conflict of interest
Krzysztof Pietrzkiewicz	Development panel	Doctor	Poznan University of Medical Sciences	Poland	Paediatric Anaesthesia, Paediatric Intensive Care, Clinical Pharmacology, Pain Management in Paediatrics		None
Francesca Sperotto	Development panel	Doctor	Boston Children's Hospital	USA	Clinical outcome research, Pediatric Cardiology outcome research, Clinical trials,<br>Systematic reviews and meta-analysis - methodology		None
Benjamin Wyness	Development panel	Pharmacist	Cambridge University Hospital of NHS Foundation Trust	UK	NICU, PICU, Pain, Sedation		None
Nilüfer Yalındağ	Development panel	Doctor	Marmara University School of Medicine	Turkey	Pain, Sedation, PICU		None
