# Supplementary material for: Pain, Agitation, Delirium, and Iatrogenic Withdrawal Syndrome Management in Children Who Are Critically Ill: Protocol for a European Clinical Practice Guideline Using the Grading of Recommendations Assessment, Development, and Evaluation Approach
Source: JMIR Res Protoc. 2025 Sep 8;14:e67930. doi: 10.2196/67930 (PMC12455155; doi:10.2196/67930)
Supplement: Multimedia Appendix 9 [file resprot_v14i1e67930_app9.pdf]

Summary recommendation	Clinical expert agreement (n=19), n (%)	Consensus meeting decisions (n=14)	Transformation into a research question	Patient and family partner agreement (n=10), n (%)
**Assessment**				
Children as young as aged 4 y but certainly from the age of 6 y should be asked to self-report their pain using the VAS[a] or NRS[b] whenever possible	18 (95)	Recommended instruments were removed in the transformation step; evidence will dictate which are recommended	What validated pain self-report scales are recommended for use in children aged 4-18 y?	10 (100)
Use of age-appropriate scales to assess pain in noncommunicative children who are critically ill (the FLACC[c], COMFORT-B[d], or MAPS[e] scales)	19 (100)	This summary recommendation was combined with the one in the following row as some instruments assessed both conditions. Recommended instruments were removed in the transformation step; evidence will dictate which are recommended.	What validated pain, sedation, and analgosedation scales are recommended for use in noncommunicative children who are critically ill?	10 (100)
Use of age-appropriate scales to assess sedation in noncommunicative children who are critically ill (COMFORT-B)	19 (100)	This summary recommendation was combined with the one in the previous row as some instruments assessed both conditions. Recommended instruments were removed in the transformation step; evidence will dictate which are recommended.	What validated pain, sedation, and analgosedation scales are recommended for use in noncommunicative children who are critically ill?	10 (100)
Use of age-appropriate scales for monitoring delirium in children who are critically ill (CAPD[f], pCAM-ICU[g], SOS-PD[h], and PAED[i])	19 (100)	This summary recommendation was combined with the one in the following row as some instruments	What validated scales are recommended for monitoring and screening delirium or	10 (100)

		assessed both conditions. Recommended instruments were removed in the transformation step; evidence will dictate which are recommended.	IWS[j] in children who are critically ill?	
Use of age-appropriate scales to assess IWS in children who are critically ill (SOS[k] or SOS-PD and WAT-1[l])	19 (100)	This summary recommendation was combined with the one in the previous row as some instruments assessed both conditions. Recommended instruments were removed in the transformation step; evidence will dictate which are recommended.	What validated scales are recommended for monitoring and screening delirium or IWS in children who are critically ill?	10 (100)
For children with developmental delays, use specially validated measurement instruments such as the Paediatric Pain Profile or Noncommunicating Children's Pain Checklist–Revised, INRS[m], for pain assessment while considering their limitations and involving the caregivers	19 (100)	Recommended instruments were removed in the transformation step; evidence will dictate which are recommended	What validated pain scales are recommended for children with developmental delays who are critically ill?	10 (100)
**Monitoring**				
Ensure regular monitoring of analgosedation levels in pediatric patients in the PICU[n]. Increase pain assessment to every 1-2 h for patients receiving analgesic infusion. Assess and document sedation every 4-8 h or as needed based on sedation scores or the child's clinical condition.	15 (79)	Included—time frames were the issue in agreement, so these were removed; evidence will determine the appropriate frequency	What is the recommended interval or frequency for assessing analgosedation in children who are critically ill?	10 (100)
Regular and routine screening for delirium in pediatric	19 (100)	Time frames were the issue in agreement, so	What is the recommended interval	10 (100)

patients in the PICU. Assess and document every 8-12 h (at least once per shift), 24-48 h after admission, or as indicated by the child's clinical condition.		these were removed; evidence will determine the appropriate frequency	or frequency for assessing delirium in children who are critically ill?	
Regular and routine screening for IWS in pediatric patients in the PICU after 3-5 d of continuous therapy with opioids or benzodiazepines	19 (100)	Time frames were the issue in agreement, so these were removed; evidence will determine the appropriate frequency	What is the recommended interval or frequency for assessing IWS in children who are critically ill?	10 (100)
**Pain**				
As a first-line approach, IV[o] opioids, specifically morphine, should be used for the treatment of moderate to severe pain in pediatric patients who are critically ill. Morphine is the preferred opioid for managing severe pain after surgery.	17 (89)	Medication specifics were removed	Which IV opioids should be used as first-line treatment for moderate to severe pain in children who are critically ill?	10 (100)
Adjunct NSAIDs[p] (IV or oral) can be added to improve early postoperative analgesia in patients who are critically ill, particularly ibuprofen or diclofenac for children aged ≥3 y	16 (84)	Medication specifics were removed	Which adjunct NSAIDs, either IV or oral, should be used to improve early postoperative analgesia in children who are critically ill?	10 (100)
Adjunct acetaminophen (IV or oral) can improve mild postoperative pain in pediatric patients who are critically ill. It can be used from 28 wk of gestational age.	18 (95)	No discussion or changes	Which adjunct nonopioid analgesics (eg, paracetamol) should be used to improve mild postoperative pain in children who are critically ill?	10 (100)
Patient-centered pain management should be applied in the PICU with targeted therapy for each patient to decrease opioid requirements and ensure that the minimal effective dose is administered. Medications should be adjusted for poor general condition, hepatic and	16 (84)	Rejected—summary recommendation was deemed too complex for transformation, and elements would appear in the discussion of the related pain recommendations developed	Not transformed	8 (80) on rejecting

renal dysfunction, and myopathy; when using medication with the same “pathway”; and when used for >3 d.				
Identify and address potential sources of pain in children by establishing hospital-wide standards for pain prevention	13 (68)	Rejected—it was determined that this was an organizational-level implementation strategy rather than a recommendation	Not transformed	Not included
**Sedation**				
Consider adopting alpha-2 agonists as the primary sedative class in pediatric patients who are critically ill and require mechanical ventilation as the first-line approach	17 (89)	No discussion or changes	Should alpha-2 agonists be used as the first-line sedative class in children who are critically ill and on mechanical ventilation?	10 (100)
Daily sedation interruption is not suggested and should be used with caution	14 (74)	Included—the issue was with wording, and the group agreed that it was important	Should daily sedation interruption be used instead of continuous sedation until weaning in children who are critically ill?	9 (86)
In children who are difficult to sedate, consider using ketamine due to its good safety profile	15 (79)	Rejected—focus on 1 drug was too specific. The group felt that defining “difficult to sedate” was the important part of this summary recommendation, so this was added as a new research question.	Not included	Not included
**Weaning**				
Use a standardized sedation and analgesia weaning protocol, gradually reducing the dose by a maximum of 20% per day from the initial dose. Modify the weaning plan as needed and consider supplementing with alpha-2 agonists when appropriate.	18 (95)	Transformed into 2 research questions	What is the recommended rate of medication weaning in children who are critically ill as part of weaning protocols? What medications should be considered when modifying the weaning plans for	10 (100) for both questions

			children who are critically ill?	
**Delirium**				
Minimize benzodiazepine use to reduce the incidence, duration, and severity of delirium in pediatric patients who are critically ill	19 (100)	No discussion or changes	Does minimizing the use of benzodiazepines reduce the incidence, duration, and severity of delirium in children who are critically ill?	10 (100)
The routine use of haloperidol or atypical antipsychotics for the prevention of pediatric delirium is not recommended	16 (84)	No discussion or changes	Is the use of antipsychotics (either typical or atypical) recommended for the prevention of delirium in children who are critically ill?	10 (100)
In pediatric patients who are critically ill with refractory delirium, medication should be considered if nonpharmacological interventions fail to provide rapid relief, especially from agitation, restlessness, delusions, hallucinations, or risks to the patient's safety. Risperidone is the preferred choice for mild to moderate symptoms if oral administration is possible and there is sensitivity to extrapyramidal side effects. Haloperidol is the preferred choice for severe symptoms or when oral administration is not possible.	17 (89)	No discussion or changes	What medications should be used in children who are critically ill with mild, moderate, and severe delirium?	10 (100)
Weighing the risk of QT prolongation when starting antipsychotics, especially in high-risk groups and those with risk factors, and implementing monitoring through baseline electrocardiogram and routine electrolyte and QTc interval monitoring for patients	16 (84)	No discussion or changes	Is assessing baseline and serial QTc intervals necessary for patients receiving antipsychotics who are critically ill?	10 (100)

receiving haloperidol or atypical antipsychotics				
In pediatric patients at risk of or with delirium, promote parental involvement by providing clear explanations of its importance. Encourage active participation during rounds, involve them in direct patient care, and ensure their continuous presence.	19 (100)	Rejected—considered too narrow and was incorporated into the parental involvement summary recommendation (see the parental involvement category in this table)	Not included	Not included
For children at risk of delirium and those with delirium, collaborate as a multidisciplinary team to determine the most appropriate treatment based on the child's clinical presentation using interdisciplinary rounds	17 (89)	Rejected—considered too narrow for delirium alone and was instead broadened to include multidisciplinary strategies for managing all 4 conditions	What is the effect of multidisciplinary team strategies (ie, interdisciplinary rounds) on determining treatment and outcomes in pediatric patients who are critically ill based on their clinical presentation?	10 (100)
Implement delirium bundles to prevent delirium in pediatric patients, which should include promoting parental presence or voice recording; orientating the child using familiar items and functional aids; ensuring consistent nursing care with staff introductions; adapting communication using simple sentences and aids; minimizing overstimulation from noise, light, and excessive people; and establishing a structured day-night schedule. In addition, prioritize early mobilization and reserve the use of restraints as a last resort after all other safety-oriented alternatives, such as bed rails, antislip measures, and	19 (100)	Rejected—considered too narrow. Part of it is now included in the parental involvement question (see the parental involvement category in this table), whereas the rest was incorporated into a broader question on environmental measures covering all conditions (see the environmental or nonpharmacological category in this table).	Not transformed; new question created	Not included

physical supervision, have failed.				
**IWS**				
IWS should be treated with an additional bolus of the same medication that caused the symptoms	12 (63)	Accepted—wording clarified to include the treatment recommendations for sedatives and opioids	When IWS is suspected due to opioids or sedatives, what medications should be used to treat it in children who are critically ill? (1) Should alpha-2 agonists be used to manage withdrawal from sedatives? (2) Should methadone be used to manage withdrawal from opioids?	10 (100)
**Environmental or nonpharmacological**				
Optimize the PICU environment by providing earplugs and headphones to reduce noise and promote better sleep	18 (95)	It was broadened to include all 4 conditions and search for and evaluate the evidence on a wider range of environmental strategies	What is the effect of nonpharmacological interventions on managing pain, anxiety, delirium, and IWS in children who are critically ill?	10 (100)
**Organizational**				
Implement protocolized assessment of pain, sedation, delirium, and weaning (to prevent IWS) to manage these conditions and reduce adverse outcomes	18 (95)	No discussion or changes	Is protocolized management of pain, sedation, delirium, and weaning more effective than usual care in children who are critically ill?	10 (100)
Incorporate pain, sedation, and delirium management into unit guidelines and procedures and conduct regular quality audits of adherence and effectiveness along with staff training	19 (100)	Rejected—considered too broad and at the organizational level; this is part of strategies used for universal implementation and not a recommendation	Not transformed	8 (80) on rejecting
**Parental involvement**				
Parental involvement in the PICU should include promoting understanding of analgesia, sedation, and off-label drug use; informing about the risks of withdrawal	18 (95)	Removed different strategies	What is the effect of parent or caregiver strategies on managing pain, anxiety, delirium, and IWS in children who are critically ill?	10 (100)

syndrome and delirium; and facilitating presence during routine care and interventional procedures to enhance child comfort, reduce parental stress and anxiety, and improve satisfaction with care				

[a]VAS: visual analogue scale.

[b]NRS: numerical rating scale.

[c]FLACC: Face, Legs, Activity, Cry, Consolability scale.

[d]COMFORT-B:COMFORT-Behavior scale.

[e]MAPS: Multidimensional Assessment of Pain Scale.

[f]CAPD: Cornell Assessment of Pediatric Delirium.

[g]pCAM-ICU: Pediatric Confusion Assessment Method for the Intensive Care Unit.

[h]SOS-PD: Sophia Observation withdrawal Symptoms- Pediatric Delirium scale.

[i]PAED: Pediatric Anesthesia Emergence Delirium scale.

[j]IWS: iatrogenic withdrawal syndrome.

[k]SOS: : Sophia Observation withdrawal Symptoms

[l]WAT-1: Withdrawal Assessment Tool-1.

[m]INRS: individualized NRS.

[n]PICU: pediatric intensive care unit.

[o]IV: intravenous.

[p]NSAID: nonsteroidal anti-inflammatory drug.
