# Supplementary material for: Pain, Agitation, Delirium, and Iatrogenic Withdrawal Syndrome Management in Children Who Are Critically Ill: Protocol for a European Clinical Practice Guideline Using the Grading of Recommendations Assessment, Development, and Evaluation Approach
Source: JMIR Res Protoc. 2025 Sep 8;14:e67930. doi: 10.2196/67930 (PMC12455155; doi:10.2196/67930)
Supplement: Multimedia Appendix 10 [file resprot_v14i1e67930_app10.pdf]

Table.7¿New.Research.questions._prioritized.by.both.groups

Questions	Clinical experts		Steering committee	Development panel	Patient and family partners		Development panel
	Median	Mean	Initial decision	Consensus meeting 1	Median	Mean	Consensus meeting 2
Assessment							
What validated assessment scales are available for critically ill children with neurodevelopmental delays for monitoring sedation, delirium, and iatrogenic withdrawal syndrome?	7.5	7	Include	Include	6	5.8	Include
Pain							
Should fentanyl versus morphine be used to optimize patient outcomes in mechanically ventilated critically ill children?	8	7.22	Include	Include	6.5	6.33	Include
Should sufentanil versus morphine be used to optimize patient outcomes in mechanically ventilated critically ill children?	8	7.22	Include	Include	6	6.6	Include
Sedation							
What are the recommended sedation levels in critically ill children during acute, stable, and recovery phases?	8	7.56	Include	Include	9	8.13	Include
Should the goal be to keep sedation light for all critically ill children unless there is a medical reason not to (which patients should not have light sedation)?	8	8.12	Include	Include	8	8.14	Include
If the goal level of sedation cannot be reached with the first-choice medications, what other medications should be used, and in what order should they be used in critically ill children?	9	8.47	Include	Include	8	8.57	Include
should inhaled sedatives be used instead of intravenous (in the vein) sedatives for critically ill children who are hard to sedate?	7	6.28	Include	Include	7	6.71	Include
What is the best medication to bolus (to give quickly) to sedate critically ill children before procedures (like suctioning)?	7	6.35	Include	Include	8	7.14	Include
When should benzodiazepines be used for critically ill children?	6.71	7	Exclude	Panel felt this was important to include	8	8.25	Include

Questions	Clinical experts		Steering committee	Development panel	Patient and family partners		Development panel
	Median	Mean	Initial decision	Consensus meeting 1	Median	Mean	Consensus meeting 2
What is the definition for light-sedation and difficult-to-sedate in critically ill children?	8	7	Include	Include	6	6.83	Include
Analgosedation							
What is the best way for rotating analgesics (pain medications) and sedatives to manage tolerance and iatrogenic withdrawal syndrome in critically ill children?	7	7.12	Include	Include	9	8.33	Include
Delirium							
When should delirium in critically ill children be treated with a medication?	8	7.76	Include	Include	7.5	7.83	Include
Neuromuscular blocking agents							
What methods should be used to monitor muscle relaxation, sedation, and pain in critically ill children receiving neuromuscular blocking agents?	7	6.94	Include	Include	7	6.86	Include
When is using neuromuscular blocking agents indicated (continuous vs. intermittent) in critically ill children?	7	6,94	Include	Include	7	7	Include
Non-pharmacological/environmental							
What non-medication methods are good for managing pain, anxiety, delirium, and iatrogenic withdrawal syndrome in critically ill children?	8	7.17	Include	Include	7	6.86	Include
What medications help critically ill children sleep better?	8	7.11	Include	Include	7	6.88	Include
What non-medication methods help critically ill children sleep better?	8	6.74	Include	Include	7	6.38	Include
Is moving as soon as possible (early mobilization) good for improving outcomes in critically ill children?	7	7.22	Include	Include	8	7.57	Include
