# Supplementary material for: Pain, Agitation, Delirium, and Iatrogenic Withdrawal Syndrome Management in Children Who Are Critically Ill: Protocol for a European Clinical Practice Guideline Using the Grading of Recommendations Assessment, Development, and Evaluation Approach
Source: JMIR Res Protoc. 2025 Sep 8;14:e67930. doi: 10.2196/67930 (PMC12455155; doi:10.2196/67930)
Supplement: Multimedia Appendix 11 [file resprot_v14i1e67930_app11.pdf]

**Suggested new research questions**	**Comment about exclusion**
A context question around understanding short and long-term benefits and disbenefits for children that are frequently admitted to PICUs and therefore regularly treated with the medications noted	Not a new research question and is covered as part of all questions related to medications, and the aim of the guideline is to have the least impact on critically ill children
What are the good ways to use family members and caregivers for providing non-medicated sedation and relaxation in critically-ill child patients?	Not a new research question, these are covered under: *"What are some ways parents or caregivers can be involved to help manage pain, sedation, weaning, and preventing or spotting delirium in critically ill children?"*
At which stage of the weaning process the increased or continuous presence of family members or caregivers can be recommended?	
How should the amount of sedatives used be varied over the intubation period? (i.e. should there be higher levels of sedatives used initially to achieve safe sedation and then decreasing; or, slowly increasing as required)	Not a new research question, it is covered under: *"What are the recommended sedation levels in critically ill children during acute, stable, and recovery phases?"*
How readily should guidelines be followed, as opposed to using experience gained from previous admissions of a child? (i.e. tailoring approaches to individual patients)	Not a new research question and is related to: *"Is protocolized management of pain, sedation, delirium, and weaning more effective than usual care in critically ill children?"*
Managing oral medications -> pay attention to oral disorders and the size of the medication according to the child's age. Always opt for soluble formulations.	Not a question but something to consideration in any medication question related to oral medications
