# Supplementary material for: Pain, Agitation, Delirium, and Iatrogenic Withdrawal Syndrome Management in Children Who Are Critically Ill: Protocol for a European Clinical Practice Guideline Using the Grading of Recommendations Assessment, Development, and Evaluation Approach
Source: JMIR Res Protoc. 2025 Sep 8;14:e67930. doi: 10.2196/67930 (PMC12455155; doi:10.2196/67930)
Supplement: Multimedia Appendix 12 [file resprot_v14i1e67930_app12.pdf]

**Research questions for inclusion in guideline** (Total 40): Organized by question type and grouped by focus area
**Assessment and monitoring**
1. What validated pain self-report scales are recommended for use in children 4 to 18 years of age?
2. What validated analgosedation scales are recommended for use in non-communicative, critically ill children?
3. What validated scales are recommended for monitoring or screening delirium or iatrogenic withdrawal syndrome in critically ill children?
4. What validated pain scales are recommended for use in critically ill children with neurodevelopmental delays?
5. What validated scales are recommended for monitoring sedation, delirium, and iatrogenic withdrawal syndrome in critically ill children with neurodevelopmentally delays?
6. What is the recommended interval or frequency for assessing analgosedation in critically ill children?
7. What is the recommended interval or frequency for assessing delirium in critically ill children?
8. What is the recommended interval or frequency for assessing iatrogenic withdrawal syndrome in critically ill children?
**Pain**
9. Which intravenous opioids should be used as first-line treatment for moderate to severe pain in critically ill children?
10. Which adjunct non-steroidal anti-inflammatory drugs (NSAIDs), either intravenous or oral, should be used to improve early postoperative analgesia in critically ill children?
11. Which adjunct non-opioid analgesics (e.g., paracetamol) should be used to manage mild postoperative pain in critically ill children?
12. Should fentanyl versus morphine be used in mechanically ventilated, critically ill children?
13. Should sufentanil versus morphine be used in mechanically ventilated, critically ill children?
**Sedation**
14. Should alpha-2 agonists be used as the first-line sedative class in mechanically ventilated, critically ill children?
15. Should daily sedation interruption be used instead of continuous sedation until weaning in critically ill children?
16. What are the recommended sedation targets during acute, stable, and recovery phases in critically ill children?
17. Should light sedation be the goal for all critically ill children unless medically contraindicated (and if so, which patients should not receive light sedation)?
18. For critically ill children who do not achieve sedation targets with first-line agents, what medications or adjuvants should be used, and in what order of preference?
19. Should inhaled sedatives versus intravenous sedatives be used in difficult-to-sedate critically ill children?
20. What medications should be used for bolus/quickly sedate critically ill children before procedures (i.e. suctioning)?
21. When should benzodiazepines be used in critically ill children?
22. What is the definition for “light-sedation” and “difficult-to-sedate” in critically ill children?
**Weaning, analgosedation, iatrogenic withdrawal syndrome**
23. What is the recommended rate for weaning analgesics and sedatives in critically ill children as part of weaning protocols?
24. What medications should be considered when modifying the weaning plans for critically ill children?
25. What are the effects of rotating analgesics and sedatives on tolerance and withdrawal in critically ill children?

26. When iatrogenic withdrawal syndrome is suspected due to opioids or sedatives, what medications should be used for treatment in critically ill children? a) Should alpha2-agonists be used to manage withdrawal from sedatives? b) Should methadone be used to manage withdrawal from opioids?
**Delirium**
27. Does minimizing benzodiazepine use reduce the incidence, duration, and severity of delirium in critically ill children?<br>28. Is the use of antipsychotics (typical or atypical) recommended for the prevention of delirium in critically ill children?<br>29. What medications should be used in critically ill children to treat mild, moderate and severe delirium in critically ill children?<br>30. Is baseline and serial QTc intervals monitoring necessary for critically ill children receiving antipsychotics?
31. When should pharmacological agents be used to treat delirium in critically ill children?
**Neuromuscular blocking agents**
32. What approaches should be used to monitor muscle relaxation, sedation, and pain in critically ill children receiving neuromuscular blocking agents?<br>33. When is the use of NMBAs (continuous vs. intermittent) indicated in critically ill pediatric patients?
**Other: Parents, non-pharmacological, sleep, early mobilization, protocolization, multidisciplinary**
34. What is the effect of parent or caregiver strategies on managing pain, anxiety, delirium, and iatrogenic withdrawal syndrome in critically ill children?<br>35. What is the effect of non-pharmacological interventions on managing pain, anxiety, delirium, and iatrogenic withdrawal syndrome in critically ill children?
36. What pharmacological options should be used to promote sleep in critically ill children?<br>37. What non-pharmacological options should be used to promote sleep in critically ill children?<br>38. What is the effect of early mobilization interventions on managing pain, anxiety, delirium, and iatrogenic withdrawal syndrome in critically ill children?
39. Is protocolized management of pain, sedation, delirium, and weaning more effective than usual care in critically ill children?<br>40. What is the effect of multidisciplinary team strategies (i.e., interdisciplinary rounds) on treatment decisions and outcomes in critically ill children?

Summary recommendation, transformed into research questions

New research questions
