# Supplementary material for: Pain, Agitation, Delirium, and Iatrogenic Withdrawal Syndrome Management in Children Who Are Critically Ill: Protocol for a European Clinical Practice Guideline Using the Grading of Recommendations Assessment, Development, and Evaluation Approach
Source: JMIR Res Protoc. 2025 Sep 8;14:e67930. doi: 10.2196/67930 (PMC12455155; doi:10.2196/67930)
Supplement: Multimedia Appendix 13 [file resprot_v14i1e67930_app13.pdf]

Title	Reason for exclusion
Guidance on sedation and anesthesia protocols for diagnostic and therapeutic procedures (I) [1]	Wrong outcome
Thorough assessment of pain is the key to diagnosis and management [2]	Wrong publication type
Agitated Patients in the Intensive Care Unit: Guidelines for Causal Rather Than Symptomatic Treatment are Warranted [3]	Wrong population
Guidelines on postoperative delirium: Where do we go from here? [4]	Wrong publication type
Position Statement: Authorized Agent Controlled Analgesia [5]	Wrong outcome
[Psychosocial factors in pain and pain management : A statement] [6]	Wrong publication type
Society of Anesthesia and Sleep Medicine Position Paper on Patient Sleep During Hospitalization [7]	Wrong outcome
Actions to Avoid in Pain Management and Sedoanalgesia Procedures in Pediatric Emergencies [8]	Wrong publication type
EAHP-EANM joint statement [9]	Wrong publication type
Updated sedative basics guide: everything you need to know about sedatives in clinical practice [10]	Wrong population
Evidence Summary. Delirium in Children: Prevention and Management in Postoperative Care [11]	Wrong publication type
2021 KSCCM clinical practice guidelines for pain, agitation, delirium, immobility, and sleep disturbance in the intensive care unit [12]	Wrong population
Analgo-sedation in Patients on Non-invasive Mechanical Ventilation: Need for Guideline Recommendation [13]	Wrong publication type
Developing expert international consensus statements for opioid-sparing analgesia using the Delphi method [14]	Wrong publication type
Position Statement: End the Use of the Term "Excited Delirium" [15]	Wrong publication type
[Expert consensus on late stage of critical care management] [16]	Wrong outcome
Guidance on sedation and anesthesia protocols for iagnostic and therapeutic procedures (III) [17]	Wrong outcome
Delirium Position Statement [18]	Wrong population
Guidance on sedation and anesthesia protocols for diagnostic and therapeutic procedures (II). [19]	Wrong outcome
Chinese expert consensus on sedation and analgesia for neurocritical care patients. [20]	Wrong population

References

1. Chinese Society of Anesthesiology Task Force. Guidance on sedation and anesthesia protocols for diagnostic and therapeutic procedures (I). 诊疗性操作的镇静与麻醉方案指导意见(一). 2023;43(6):641-7. Doi:10.3760/cma.j.cn131073.20230606.00601
2. Azeem Z. Thorough assessment of pain is the key to diagnosis and management. BJOG. 2023;130(8):990. Doi:10.1111/1471-0528.17435
3. Azimaraghi O, Smith V, Sauer WJ, Alpert JE, Eikermann M. Agitated Patients in the Intensive Care Unit: Guidelines for Causal Rather Than Symptomatic Treatment are Warranted. J Intensive Care Med. 2023;38(2):238-40. Doi: 10.1177/08850666221138234
4. Bruder N, Chew M. Guidelines on postoperative delirium: Where do we go from here? Eur J Anaesthesiol. 2024;41(2):79-80. Doi: 10.1097/EJA.0000000000001935

5. Czarnecki M, Cooney MF, Wuhrman E. ASPMN Position Statement: Authorized Agent Controlled Analgesia. Pain Manag Nurs. 2024;25(3):209-10. Doi: 10.1016/j.pmn.2024.03.016
6. Eich W, Diezemann-Prossdorf A, Hasenbring M, Huppe M, Kaiser U, Nilges P, et al. [Psychosocial factors in pain and pain management : A statement]. Schmerz. 2023;37(3):159-67. Doi: 10.1007/s00482-022-00633-1
7. Hillman DR, Carlucci M, Charchaflieh JG, Cloward TV, Gali B, Gay PC, et al. Society of Anesthesia and Sleep Medicine Position Paper on Patient Sleep During Hospitalization. Anesth Analg. 2023;136(4):814-24. Doi: 10.1213/ANE.0000000000006395
8. Khodayar-Pardo P, Miguez-Navarro MC, Martin Espin I, Analgesia, Sedation Working Group of the Spanish Society of Pediatric Emergency M. Actions to Avoid in Pain Management and Sedoanalgesia Procedures in Pediatric Emergencies. Pediatr Emerg Care. 2024;40(4):e23-e29.Doi: 10.1097/PEC.0000000000003053
9. Kohl S. EAHP-EANM joint statement. Eur J Hosp Pharm. 2023;30(2):122.Doi: 10.1136/ejhpharm-2023-003717
10. Lee OH. Updated sedative basics guide: everything you need to know about sedatives in clinical practice. J of Kor Med Assoc. 2024;67(4):285-95.Doi:10.5124/jkma.2024.67.4.285
11. Marin, T, Johal,, J. Evidence Summary. Delirium in Children: Prevention and Management in Postoperative Care. The JBI EBP Database. 2023; JBIES-287-4.
12. Seo Y, Lee HJ, Ha EJ, Ha TS. 2021 KSCCM clinical practice guidelines for pain, agitation, delirium, immobility, and sleep disturbance in the intensive care unit. Acute Crit Care. 2023;38(1):149.Doi: 10.4266/acc.2022.00094.e1
13. Shrinath V, Marwah V, Jyothis MC. Analgo-sedation in Patients on Non-invasive Mechanical Ventilation: Need for Guideline Recommendation. Indian J Crit Care Med. 2024;28(3):317-8. Doi:10.5005/jp-journals-10071-24642
14. Sng DD, Uitenbosch G, de Boer HD, Carvalho HN, Cata JP, Erdoes G, et al. Developing expert international consensus statements for opioid-sparing analgesia using the Delphi method. BMC Anesthesiol. 2023;23(1):62. Doi: 10.1186/s12871-023-01995-4
15. Stolbach AI, Dargan PI, Greller HA, Hamilton RJ, Johnson-Arbor K, Murray BP, et al. ACMT Position Statement: End the Use of the Term "Excited Delirium". J Med Toxicol. 2023;19(3):310-2. Doi: 10.1007/s13181-023-00944-4
16. Tang B, Chen WJ, Jiang LD, Zhu SH, Song B, Chao YG, et al. [Expert consensus on late stage of critical care management]. Zhonghua Nei Ke Za Zhi. 2023;62(5):480-93. Doi: 10.3760/cma.j.cn112138-20221005-00731
17. Wang E. Guidance on sedation and anesthesia protocols for iagnostic and therapeutic procedures (III). 诊疗性操作的镇静与麻醉方案指导意见(III). 2023;43(6):658. Doi:10.3760/cma.j.cn131073.20230606.00603
18. Yager M, Clark BR, Gulley N, Denny DL. Delirium Position Statement. Orthop Nurs. 2023;42(3):147-50. Doi: 10.1097/NOR.0000000000000940
19. Yunqi L. Guidance on sedation and anesthesia protocols for diagnostic and therapeutic procedures (II). 诊疗性操作的镇静与麻醉方案指导意见(二). 2023;43(6):648-57. Doi:10.3760/cma.j.cn131073.20230606.00602
20. Zhang L, Liu S, Wang S, Zhou JX, National Center for Healthcare Quality Management in Neurological D, Chinese Society of Critical Care M, et al. Chinese expert consensus on sedation and analgesia for neurocritical care patients. Chin Med J (Engl). 2024;137(11):1261-3.Doi: 10.1097/CM9.0000000000003084
