# Supplementary material for: Pain, Agitation, Delirium, and Iatrogenic Withdrawal Syndrome Management in Children Who Are Critically Ill: Protocol for a European Clinical Practice Guideline Using the Grading of Recommendations Assessment, Development, and Evaluation Approach
Source: JMIR Res Protoc. 2025 Sep 8;14:e67930. doi: 10.2196/67930 (PMC12455155; doi:10.2196/67930)
Supplement: Multimedia Appendix 14 [file resprot_v14i1e67930_app14.pdf]

Society, year (ref)	Title	Country	Language	Type of CPG	Version	Population (setting)	Conditions				System for certainty of evidence	System for strength of recommend-ation	Patients on panel	Used AGREE II
							P	S	D	W				
Pediatric Intensive Care Sedation-Analgesia and Muscle Relaxant Group , 2024 [1]	Sedation-analgesia, muscle relaxant applications in pediatric intensive care units and guidelines for the management and environment optimization of clinical statements such as withdrawal, delirium developed during these applications	Turkey	English	Guideline	New	Pediatrics (PICU)	■	■	■	■	States GRADE but not defined how it was used for either certainty of evidence or strength of recommendations		No	No
Chinese Medical Association and Chinese Medical Association Emergency Medicine, 2024 [2]	Expert consensus on analgesia and sedation for children in pediatric intensive care units of China	China	Chinese	Consensus	Third revision	Pediatrics (PICU)	■	■	□	□	NI	NI	No	No

Conditions: P = pain, S = sedation, D = delirium, W = iatrogenic withdrawal syndrome
■ = explicit recommendation related to condition
□ = implicit, included as part of the discussion in the main body of the text
NI = not indicated
PICU = pediatric intensive care unit

1. Bozan G, Koçkuzu E, Korulmaz A, Altuğ Ü, Yıldızdaş D. Sedation-analgesia, muscle relaxant applications in pediatric intensive care units and guidelines for the management and environment optimization of clinical statements such as withdrawal, delirium developed during these applications. Journal of Pediatric Emergency and Intensive Care Medicine (Turkey). 2024

2. Subspecialty Group of Emergency Medicine tSoPCMA, Subspecialty Group of Pediatrics tSoEMCMA, Editorial Board CJoP. [Expert consensus on analgesia and sedation for children in pediatric intensive care units of China (2024)]. Zhonghua Er Ke Za Zhi. 2024;62(3):196-203.10.3760/cma.j.cn112140-20231130-00400
