# Supplementary material for: Pain, Agitation, Delirium, and Iatrogenic Withdrawal Syndrome Management in Children Who Are Critically Ill: Protocol for a European Clinical Practice Guideline Using the Grading of Recommendations Assessment, Development, and Evaluation Approach
Source: JMIR Res Protoc. 2025 Sep 8;14:e67930. doi: 10.2196/67930 (PMC12455155; doi:10.2196/67930)
Supplement: Multimedia Appendix 15 [file resprot_v14i1e67930_app15.pdf]

CPGs \ AGREE II items	Dom 1			Dom 2			Dom 3								Dom 4			Dom 5				Dom 6		Overall		Quality
	1	2	3	4	5	6	7	8	9	10	11	12	13	14	15	16	17	18	19	20	21	22	23	Overall	Rec.	Quality
Pediatric Intensive Care Sedation-Analgesia and Muscle Relaxant Group , 2024 [1]	6	3	6	2	1	5	1	1	1	2	3	2	1	1	3	3	2	1	1	1	1	2	2	2	No	Low
Chinese Medical Association and Chinese Medical Association Emergency Medicine, 2024 [2]	6	2	4	3	1	4	2	2	2	1	2	2	1	3	2	3	2	1	2	1	1	1	2	2	No	Low

Dom = domain, rec = recommend, YWM = yes with modifications

1. Bozan G, Koçkuzu E, Korulmaz A, Altuğ Ü, Yıldızdaş D. Sedation-analgesia, muscle relaxant applications in pediatric intensive care units and guidelines for the management and environment optimization of clinical statements such as withdrawal, delirium developed during these applications. Journal of Pediatric Emergency and Intensive Care Medicine (Turkey). 2024
2. Subspecialty Group of Emergency Medicine tSoPCMA, Subspecialty Group of Pediatrics tSoEMCMA, Editorial Board CJoP. [Expert consensus on analgesia and sedation for children in pediatric intensive care units of China (2024)]. Zhonghua Er Ke Za Zhi. 2024;62(3):196-203.10.3760/cma.j.cn112140-20231130-00400
