# Supplementary material for: Pain, Agitation, Delirium, and Iatrogenic Withdrawal Syndrome Management in Children Who Are Critically Ill: Protocol for a European Clinical Practice Guideline Using the Grading of Recommendations Assessment, Development, and Evaluation Approach
Source: JMIR Res Protoc. 2025 Sep 8;14:e67930. doi: 10.2196/67930 (PMC12455155; doi:10.2196/67930)
Supplement: Multimedia Appendix 16 [file resprot_v14i1e67930_app16.pdf]

	Initial match by guideline development lead		Co-lead agreement	Final decision	
**Questions**	**Possible match (guideline – recommendation)**	**Match (guideline -recommendation)**	**Comment**	**Match**	**Unmatch**
**Assessment**					
What validated scales are recommended to monitor sedation, delirium, and iatrogenic withdrawal syndrome in neurodevelopmentally delayed critically ill children?		SARNePI - In children with developmental delay, we suggest adopting validated tools to monitor the level of sedation, the presence of delirium and withdrawal syndrome in ICU, considering their limitations and involving the caregivers [1]	Agreed	X	
**Pain**					
1. Should fentanyl versus morphine be used in mechanically ventilated critically ill children?<br>2. Should sufentanil versus morphine be used in mechanically ventilated critically ill children?	NVP - Morphine is the opioid of choice in children for controlling severe pain after major surgery. Morphine administration via continuous infusion or by PCA is preferred, in combination with paracetamol and/or NSAID. Dosage and monitoring should take into account technique, age, weight and underlying suffering [2]<br>SCCM - We recommend that IV opioids be used as the primary analgesic for treating moderate to severe pain in critically ill pediatric patients [3]<br>SARNePI - As a first-line strategy, we suggest optimizing analgesia using opiates and adopting alpha agonists as sedative agents, considering benzodiazepines a second-line [1]		Do not think any answer these questions<br><br>Agreed		X
**Sedation**					
What are the recommended sedation targets in critically ill children during acute, stable, and recovery phases?	SCCM - We suggest that all pediatric patients requiring MV be assigned a target depth of sedation using a validated sedation assessment tool at least once daily [3]<br>AWMF - In paediatric intensive care, patient-centered therapy strategies for analgesia, sedation, anxiety and delirium should be applied with individual targets of therapy goals [4]		PANDEM: does not fit phases<br>AWMF: Goes beyond just sedation<br><br>Agreed		X

	Initial match by guideline development lead		Co-lead agreement	Final decision	
**Questions**	**Possible match (guideline – recommendation)**	**Match (guideline -recommendation)**	**Comment**	**Match**	**Unmatch**
Should light sedation be the goal for all critically ill children unless medically contraindicated (which patients should not receive light sedation)?					X
In critically ill children for whom the sedation target is not achieved with first-line agents, what medications or adjuvants should be used, and in what order of preference?		SARNePI - In difficult analgesia/ sedation we suggest using ketamine, due to its good safety profile* [1] SCCM - *We suggest* consideration of adjunct sedation with ketamine in patients who are not otherwise at an optimal sedation depth [3]	Agreed	X	
Should inhaled sedatives versus intravenous sedatives be used in difficult-to-sedate critically ill children?					X
What medications should be used to bolus/quickly sedate critically ill children before procedures (i.e. suctioning)?					X
When should benzodiazepines be used in critically ill children?					X
What is the definition for light-sedation and difficult-to-sedate in critically ill children?					X
**Analgosedation**					
What are the effects of rotating analgesics and sedatives on tolerance and withdrawal in critically ill pediatric patients?					X
**Delirium**					
When should pharmacological agents be used to treat delirium in critically ill children?					X

	Initial match by guideline development lead		Co-lead agreement	Final decision	
**Questions**	**Possible match (guideline – recommendation)**	**Match (guideline -recommendation)**	**Comment**	**Match**	**Unmatch**
**Neuromuscular blocking agents (NMBAs)**					
What approaches should be used to monitor muscle relaxation, sedation, and pain in critically ill children receiving neuromuscular blocking agents?		SARNePI - We suggest monitoring the level of sedation with continuous processed EEG in patients treated with neuromuscular-blocking agents, considering the limitation and availability of the device [1]<br>SCCM - *We suggest* that train-of-four monitoring be used in concert with clinical assessment to determine depth of neuromuscular blockade<br>- Electroencephalogram-based monitoring may be a useful adjunct for assessment of sedation depth in critically ill pediatric patients receiving NMBAs [3]	Agreed	X	
When is using NMBAs indicated (continuous vs. intermittent) in critically ill pediatric patients?					X
**Environmental/non-pharmacological**					
What non-pharmacological options should be used to promote sleep in critically ill pediatric patients?		SARNePI - We recommend adopting in all paediatric patients admitted to ICU strategies to prevent sleep alterations, particularly non-pharmacologic ones (relaxing techniques, parental involvement, control of environmental factors)<br>SCCM - We suggest offering patients the use of noise reducing devices/ or behavioral changes to reduce excessive noise and therefore improve sleep hygiene and comfort, in critically ill pediatric patients. [3]<br>AWMF - A normal sleep pattern should be encouraged, especially adequate lighting, reduction of noise	Agreed	X	

	Initial match by guideline development lead		Co-lead agreement	Final decision	
**Questions**	**Possible match (guideline – recommendation)**	**Match (guideline -recommendation)**	**Comment**	**Match**	**Unmatch**
		and an adapted day-night rhythm for the patient should be taken into account [4]			
What pharmacological options should be used to promote sleep in critically ill pediatric patients?					X
What is the effect of early mobilization interventions on managing pain, anxiety, delirium, and iatrogenic withdrawal syndrome in critically ill children?	SCCM - We suggest performing EM to minimize the effects of immobility in critically ill pediatric patients [3]<br>NVvP - Consider the following interventions:<br>– Offer parental presence through rooming-in or recording parents' voices;<br>– Promote the child's orientation (staff name and function, photos, music and toys from home, calendar, whiteboard, glasses, hearing aid, dimmed light in the room at night);<br>– Have the child cared for by the same nurses as much as possible to give as much uniformity of approach/treatment as possible and ensure familiar faces. Take into account impaired attention and memory functions (simple sentences, repeating information);<br>– Avoid overstimulation from noise, draughts, light, too many people. Earplugs can be helpful here. Do not hold conversations at the bedside. If necessary, move the child to a quieter (hyperactive delirium) or more stimulating (hypoactive delirium) environment; – Mobilise the child (physiotherapy and nurses);<br>– Take developmental level into account when communicating;		Do not think these completely cover the question<br><br>Agreed		X

	Initial match by guideline development lead		Co-lead agreement	Final decision	
**Questions**	**Possible match (guideline – recommendation)**	**Match (guideline -recommendation)**	**Comment**	**Match**	**Unmatch**
	– Improve day-night rhythm by offering activities, changing daylight, among other things;<br>– Support dyspraxia, dysphasia and other factors that make communication difficult with aids (writing pad, pointer card, electronic resources) [5]				
**Multidisciplinary**					
What is the effect of multidisciplinary team strategies (i.e., interdisciplinary rounds) on determining treatment and outcomes in critically ill pediatric patients based on their clinical presentation?					X

AWMF: Arbeitsgemeinschaft der Wissenschaftlichen Medizinischen Fachgesellschaften, (AWMF—the Association of the Scientific Medical Societies); **NVvP:** Nederlands Vereniging voor Psychiatrie; **NVP:** Dutch Society of Anaesthesiology; **SARNePI:** Italian Society of Neonatal and Pediatric Anesthesia and Intensive Care; **SCCM:** Society of Critical Care Medicine

References

1. Amigoni A, Conti G, Conio A, Corno M, Fazio PC, Ferrero F, et al. Recommendations for analgesia and sedation in critically ill children admitted to intensive care unit. Journal of Anesthesia, Analgesia and Critical Care. 2022;2(1):9.10.1186/s44158-022-00036-9
2. Nederlandse Vereniging voor Anesthesiologie. Richtlijn - Postoperatieve pijn2012.
3. Smith HAB, Besunder JB, Betters KA, Johnson PN, Srinivasan V, Stormorken A, et al. 2022 Society of Critical Care Medicine Clinical Practice Guidelines on Prevention and Management of Pain, Agitation, Neuromuscular Blockade, and Delirium in Critically Ill Pediatric Patients With Consideration of the ICU Environment and Early Mobility. Pediatr Crit Care Med. 2022;23(2):e74-e110.10.1097/PCC.0000000000002873
4. AWMF o. S3-Leitlinie: Analgesie, Sedierung und Delirmanagement in der Intensivmedizin (DAS-Leitlinie 2020) [Available from: https://www.awmf.org/leitlinien/detail/ll/001-012.html.
5. Schieveld JNM, de Graeff-Meeder E, Kalverdijk L, Gerver J, Knoester H, de Neef M, et al. Multidisciplinaire richtlijn pediatrisch delier. Utrecht: De Tijdstroom; 2014.
