# Supplementary material for: Pain, Agitation, Delirium, and Iatrogenic Withdrawal Syndrome Management in Children Who Are Critically Ill: Protocol for a European Clinical Practice Guideline Using the Grading of Recommendations Assessment, Development, and Evaluation Approach
Source: JMIR Res Protoc. 2025 Sep 8;14:e67930. doi: 10.2196/67930 (PMC12455155; doi:10.2196/67930)
Supplement: Multimedia Appendix 17 [file resprot_v14i1e67930_app17.pdf]

**Clinical experts (mean, median)**	**Patient and family partners (mean, median)**
Question 1: *Should sufentanil versus morphine be used for optimizing patient outcomes in mechanically ventilated critically ill children?*	
1. Incidence of withdrawal (8, 8.5)<br>2. Proportion of pain scores within range (8, 8)<br>3. Length of mechanical ventilation (8, 7.75)<br>4. Pain intensity (7.65, 8)<br>5. Incidence of withdrawal (7.6, 8)	1. Incidence of withdrawal (8.4, 8)<br>2. Unplanned extubation (8.2, 8)<br>3. Length of mechanical ventilation (8.2, 8)<br>4. Satisfation (patient/family) (8.17, 8.5)<br>5. Long-term outcomes after PICU stay (8, 8)
Question 2: *Should fentanyl versus morphine be used for optimizing patient outcomes in mechanically ventilated critically ill children?*	
1. Incidents of withdrawal 7.84, 8)<br>2. Pain intensity (7.84, 8)<br>3. Length of mechanical ventilation (7.68, 8)<br>4. Length of PICU stay (7.63, 8)<br>5. Incidents of delirium (7.53, 7)	1. PICU mortality (8.71, 9)<br>2. Unplanned extubation (8.4, 8)<br>3. Length of mechanical ventilation (8.17, 8.5)<br>4. Satisfation (patient/family) (8,8)<br>5. Long-term outcomes after PICU stay (8,8)
Question 3: *Should inhaled versus intravenous sedatives be used for difficult to sedate critically ill children?*	
1. Length of mechanical ventilation (8, 8)<br>2. Incidence of delirium (7.95, 8)<br>3. Proportion of sedation scores in target range (7.9, 8)<br>4. Level of sedation (7.79, 8)<br>5. PICU mortality (7.7, 8)	1. PICU mortality (8.67, 9)<br>2. Incidents of withdrawal (8.5, 9)<br>3. Incidents of delirium (8.5, 8,75)<br>4. Unplanned extubation (8, 8)<br>5. Proportion of sedation scores in target range (7.8, 8)
