# Supplementary material for: Pain, Agitation, Delirium, and Iatrogenic Withdrawal Syndrome Management in Children Who Are Critically Ill: Protocol for a European Clinical Practice Guideline Using the Grading of Recommendations Assessment, Development, and Evaluation Approach
Source: JMIR Res Protoc. 2025 Sep 8;14:e67930. doi: 10.2196/67930 (PMC12455155; doi:10.2196/67930)
Supplement: Multimedia Appendix 8 [file resprot_v14i1e67930_app8.pdf]

PHASE 1: SET-UP

Action 5: Patient and family consultation on scope - results

		Individual patient and family partner data										
		**EN1**	**EN2**	**EN3**	**EN3**	**EN4**	**EN5**	**EN6**	**FR1**	**FR2**	**FR3**	**% agreement**
**Population:** Critically ill children aged 0 to 18 years in pediatric intensive care units as the primary setting, but including those awaiting admission(e.g., emergency department or transport)	Accept	1	1	1	1	1	1		1	1		**80%**
	Accept with modifications							1			1	**20%**
	Reject											0%
**Conditions to be covered in the guideline**: Pain, agitation/sedation, delirium, and iatrogenic withdrawal syndrome	Accept	1	1	1	1	1	1	1	1	1	1	**100%**
	Accept with modifications											0%
	Reject											0%
**Purpose:** To develop a high-quality, evidence-based clinical practice guideline tailored to the European context. This guideline will incorporate the perspectives of patients, families, and healthcare professionals. It aims to improve the management of these interrelated conditions (pain, sedation, delirium and iatrogenic withdrawal syndrome) for critically ill children in pediatric intensive care units by providing actionable, trustworthy, and credible recommendations	Accept	1	1	1	1	1	1	1	1	1		**90%**
	Accept with modifications										1	**10%**
	Reject											0%
**Audience:** The guideline is intended for healthcare professionalsworking in pediatric intensive care units, including doctors, nurses,pharmacists, and other relevant allied health practitioners. It will also serveas a resource for patients, families, and caregivers	Accept		1	1	1	1	1	1	1	1		**80%**
	Accept with modifications										1	**10%**
	Reject	1										**10%**
